# Genetically-Encoded Phase Separation Sensors Enable High-Fidelity Live-Cell Probing of Biomolecular Condensates

**DOI:** 10.1021/acssensors.4c02851

**Published:** 2025-02-23

**Authors:** Alexa Regina Chua Avecilla, Jeremy Thomas, Felipe Garcia Quiroz

**Affiliations:** Wallace H. Coulter Department of Biomedical Engineering, Georgia Institute of Technology and Emory University, Atlanta, Georgia 30322, United States

**Keywords:** intrinsically disordered proteins, biomolecular condensates, membraneless organelles, liquid−liquid phase separation, live-cell imaging

## Abstract

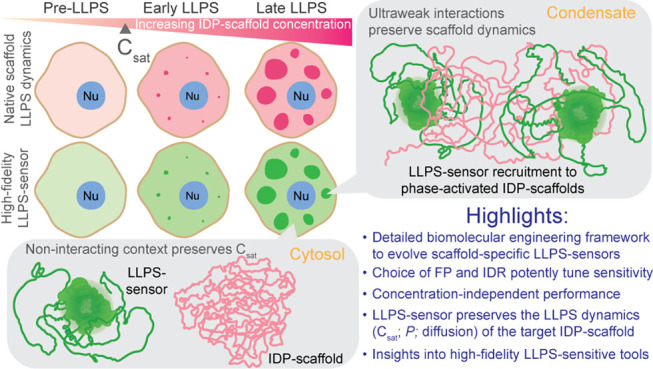

Biomolecular condensates are membraneless compartments with enigmatic roles across intracellular phenomena. Intrinsically disordered proteins (IDPs) often function as condensate scaffolds, fueled by liquid–liquid phase separation (LLPS) dynamics. Intracellular probing of condensates relies on live-cell imaging of IDP-scaffolds tagged with fluorescent proteins. Conformational heterogeneity in IDPs, however, renders them uniquely susceptible to artifacts from tagging. Probing epidermal condensates in skin, we recently introduced genetically-encoded LLPS-sensors that circumvent the need for molecular-level tagging of skin IDPs. Departing from subcellular tracking of IDP-scaffolds, LLPS-sensors report on the assembly and liquid-like dynamics of their condensates. Here, we demonstrate biomolecular approaches for the evolution and tunability of epidermal LLPS-sensors and assess their impact in the early and late stages of intracellular phase separation. Benchmarking against scaffold-bound fluorescent reporters, we discovered that tunable ultraweak scaffold–sensor interactions uniquely enable the sensitive and innocuous probing of nascent and established biomolecular condensates. Our LLPS-sensitive tools pave the way for the high-fidelity intracellular probing of IDP-governed biomolecular condensates across biological systems.

Biomolecular condensates recently emerged as ubiquitous intracellular membraneless compartments with enigmatic liquid-like properties.^1−5^ These incompletely understood condensates orchestrate a wide range of vital molecular mechanisms at cell and tissue levels.^6−9^ In addition to roles in homeostasis and stress response, aberrant biomolecular condensates are implicated in neurodegenerative diseases^5,10−12^ and cancer.^12−14^ Intrinsically disordered proteins (IDPs) and large intrinsically disordered regions (IDRs) underpin the formation and liquid-like dynamics of numerous intracellular condensates.^1,4,15,16^ They function as condensate “scaffolds”, driven by a concentration-dependent process of liquid–liquid phase separation (LLPS) or related phase transitions.^17^ Above a specific concentration unique to the IDP-scaffold, known as the saturation concentration (*C*_sat_), they segregate into two distinct phases: a dense phase of IDP-rich droplets and a dilute phase of dispersed IDP-scaffolds.^2,4^ In addition to defining condensate self-assembly dynamics, IDP-scaffolds may recruit “client” biomolecules through specific binding domains or weak IDP-IDP interactions,^3,18,19^ expanding condensate composition and functionality.^20,21^

The underlying LLPS dynamics are sensitive to environmental and molecular-level perturbations. Two salient examples are post-translational modifications of IDP-scaffolds and physiological fluctuations in pH and ions, which dramatically alter condensate assembly dynamics and material properties.^22,23^ Probing these functional dynamics intracellularly requires live-cell methods. State-of-the-art approaches involve the tagging of IDP-scaffolds with fluorescent proteins for live-cell imaging, or with enzymes for proximity-dependent biotinylation and proteomics.^3,4^ Live imaging probes the biophysical properties of intracellular condensates, while proximity proteomics enables in situ and time-dependent analyses of their biomolecular composition.^24,25^

Challenging these live-cell efforts, the molecular-level tagging of native IDP-scaffolds risks unpredictably altering their intracellular localization and underlying LLPS dynamics,^7,26,27^ This is in part because IDP-scaffolds functionally exploit high-entropy conformational dynamics that are sensitive to their molecular neighborhood.^28−30^ For neurodegeneration-relevant IDP assemblies, tagging with fluorescent proteins for live imaging has been shown to alter their composition, ultrastructure and toxicity.^31−33^ Furthermore, IDP-scaffolds may be regulated *in vivo* through proteolysis, limiting the utility of N-terminal or C-terminal protein tags. Filaggrin (FLG) exemplifies this regulation, undergoing N-terminal processing in the skin soon after condensate assembly.^7^ Through the lens of bioengineering, the immunogenicity of fluorescent protein tags^34,35^ threatens clinical translation of synthetic IDP-scaffolds and synthetic condensates^36^ in human cells.

We currently lack tools for high-fidelity live-cell probing of intracellular condensates without scaffold-level tagging. Emerging approaches such as label-free microscopy coupled with deep learning appear promising for the identification of solid-like neuropathological IDP aggregates in cell monolayers.^33^ We suspect that related approaches may also succeed with a subset of IDP-driven condensates in cultured cells. Even with sustained progress in label-free methods, biomolecular tools will be needed to achieve high-resolution and innocuous probing of condensate dynamics at biophysical and biochemical levels.

In beginning to address this challenge, we recently introduced fluorescent IDP-based LLPS-sensors that are sensitive to the ultraweak intermolecular interactions that drive the formation and LLPS dynamics of epidermal condensates.^7^ We showed that live-cell probing of sensor signal within epidermal condensates in keratinocytes mirrored expected changes in their liquid-dynamics. In mice genetically engineered to express these epidermal LLPS-sensors and other subcellular markers, we unearthed previously enigmatic keratohyalin granules (KGs) as liquid-like epidermal condensates whose assembly and pH-triggered disassembly drive skin barrier formation.^7^ Despite excitement in the IDP field for the underlying LLPS-sensors,^37,38^ this seminal work prioritized skin biology questions, and did not dwell on the overall design, evolution and concentration-dependent performance of LLPS-sensors.

To advance the understanding and development of LLPS-sensitive tools, here we pursue new quantitative approaches to fully dissect the biomolecular engineering and properties of epidermal LLPS-sensors at two new levels: (1) their sensitive marking of liquid-like epidermal condensates (i.e., high signal-to-noise ratio for live-cell approaches), and (2) the impact of LLPS-sensors on early and late stages of intracellular phase separation. Excitingly, our new experiments and data demonstrate two notable properties of LLPS-sensors: (i) highly tunable range of sensitivity, and (ii) innocuous probing of concentration-dependent intracellular LLPS dynamics. Benchmarking our top-performing epidermal LLPS-sensor against a scaffold-bound fluorescent reporter-client, we discovered that ultraweak scaffold-sensor interactions are key to the high-fidelity probing of nascent and established biomolecular condensates. Together with our detailed biomolecular framework to engineer scaffold-specific LLPS-sensors, our new findings demonstrate a path toward rigorous intracellular probing of IDP-governed biomolecular condensates across biological systems.

## Experimental Section

### Sequence-Level Prediction of Disorder and LLPS-Relevant Regions

To profile areas of intrinsic disorder in FLG, FLG-like scaffolds and engineered sensor constructs, we used DISOPRED3^39^ from the University College London PSIPRED Workbench (http://bioinf.cs.ucl.ac.uk/psipred/). Scores above 0.5 were considered predictive of intrinsic disorder. To expose LLPS-relevant regions (i.e., those rich in features involved in LLPS), we calculated droplet-promoting scores using FuzDrop^8^ (https://fuzdrop.bio.unipd.it/predictor). Generally, regions with scores at or above 0.60 are assumed to be part of droplet-promoting regions.^8^ In our interpretation of this tool, which tends to overestimate the LLPS propensity of IDPs, we considered scores above 0.6 as segments rich in LLPS-relevant sequence features. When comparing the percent sequence identity of r8 and IDP-sensing domains, we aligned the relevant sequences using Clustal Omega.^40^ To gage conformational dynamics, we generated AlphaFold2 predictions using ColabFold and colored the resulting structures using pLDDT scores as proxy for intrinsic disorder.^41,42^ In select cases (e.g., Figure S5), we also employed homology modeling with MODELER.^43^ For proteins larger than 800 residues in length, we aligned and combined overlapping AlphaFold2 models using Chimera.^44^ Protein structures were rendered with PyMOL (Version 2.5.7 Schrödinger, LLC).

### Synthesis of Repetitive DNAs Encoding FLG-like IDP-Scaffolds

To assemble FLG-like (r8)_n_ IDP-scaffolds, we used recursive directional ligation by plasmid-reconstruction (PRe-RDL) with a modified pET-24a(+) vector featuring a Gly-stop–stop–stop sequence as previously described.^7^ We previously reported successful iterative rounds of PRe-RDL to build genes with two, four or 8 repeats of r8 —with and without the C-terminal tail domain of human FLG. For IDP-scaffolds designed to interact with dTEVp (as in Figure 6), we used PRe-RDL to add the short cTEV domain to the C-terminus of (r8)_2_, resulting in (r8)_4_ constructs with two dTEVp-binding sites: a C-Terminal cTEV site and one internal cTEV linking two (r8)_2_ repeats. For mammalian expression, we subcloned all fully assembled repeat genes into published pMAX vectors (Amaxa) encoding mRFP1, mRFP1-cTEV, H2BGFP-P2A-mRFP1 or H2BGFP-P2A-mRFP1-cTEV.^7^ Fusion of our cTEV-containing (r8)_n_ genes to constructs with mRFP1-cTEV resulted in IDP-scaffolds with either one or three cTEV sites. To build genes encoding IDP-scaffolds with equimolar coexpression of LLPS-sensor or client (related to Figure 6A), we modified our published pMAX vectors encoding H2BGFP-P2A-mRFP1-tagged FLG-like IDP-scaffolds to replace the H2BGFP domain with sequence-verified genes encoding Sensor A or a sfGFP-dTEVp client. See Table S4 for protein sequences of all IDP-scaffold constructs. We previously validated the equimolar synthesis of individual P2A-linked proteins by examining H2BRFP-(P2A)-H2BGFP and H2BGFP-(P2A)-H2BRFP constructs.^7^

### Synthesis of LLPS-Sensor Variants and dTEVp-Based Client

We previously reported the synthesis of mammalian-optimized genes encoding three candidate IDP-sensing domains (r8, ir8H2, and ieFLG1; see Table S1 for sequence details), and four candidate fluorescent protein tags: sfGFP and three supercharged variants of (n20GFP, p15GFP, and p15GFPkv)—all featuring a C-terminal short nuclear export signal (LELLEDLTL).^45^ Using compatible restriction sites in our library of pMAX vectors, we assembled genes that fused each candidate IDP-sensing domain to each fluorescent protein. The pMAX vector encoding the sfGFP-tagged dTEVp client (see Table S3 for sequence details) was previously reported.^7^ For constructs incorporating P2A domains, we PCR-amplified Sensor A or sfGFP-dTEVp, adding compatible restriction sites to clone them into our published H2BGFP-(P2A)-H2BRFP vector, replacing the parent H2BGFP domain. These sequence-verified constructs were used directly (Figure 4A,B) or as cloned genes for the synthesis of constructs with P2A-linking of Sensor/Client and IDP-scaffolds as described above (related to Figure 6A).

### Quantitative Assessment of LLPS-Sensor Performance

To analyze the intracellular behavior of FLG-like IDP-scaffolds and related tools, we transfected genes of interest into HaCATs and performed live-cell imaging as previously described.^7^ Briefly, using spinning disk confocal microscopy, we generated max intensity projections to quantify key LLPS-relevant metrics. Specifically, using ImageJ we measured the extent of intracellular LLPS by an IDP-scaffold as the percentage of total (background-corrected) fluorescent signal residing within spherical condensates. Similarly, we quantified the partition coefficient of both IDP-scaffolds and our tools (LLPS-sensor designs and a ligand-type client) as the ratio of the (background-corrected) fluorescent signal intensity within condensates to the signal in the cytosol. For cells with condensates, the background-corrected cytosolic signal of IDP-scaffolds served as a proxy for the saturation concentration (*C*_sat_). When possible, we estimated intracellular concentration using H2B reporter signal adjusted by total cell area to sensitively and consistently measure concentration across divergent proteins of interest. Whenever we observed a concentration-dependent increase in the extent of LLPS (e.g., Figure 6C), we applied a logistic fit [*y* = (−100/(1 + (*x*/*x*_0_)^P^)) + 100], as expected for a phase transition^7^ using OriginPro.

### Statistical Analyses

Statistical significance indicates that we rejected, with confidence greater than 0.05 (i.e., *p* < 0.05), the null hypothesis that the difference in the mean values between two data sets equaled zero. To perform this hypothesis testing, we ran two-sample *t* tests using OriginPro. In all cases, we verified that the statistical differences did not depend on the assumption of equal variance (Welch-correction) between samples.

## Results

### Concept and Evolution of LLPS-Sensors

**Figure 1:**
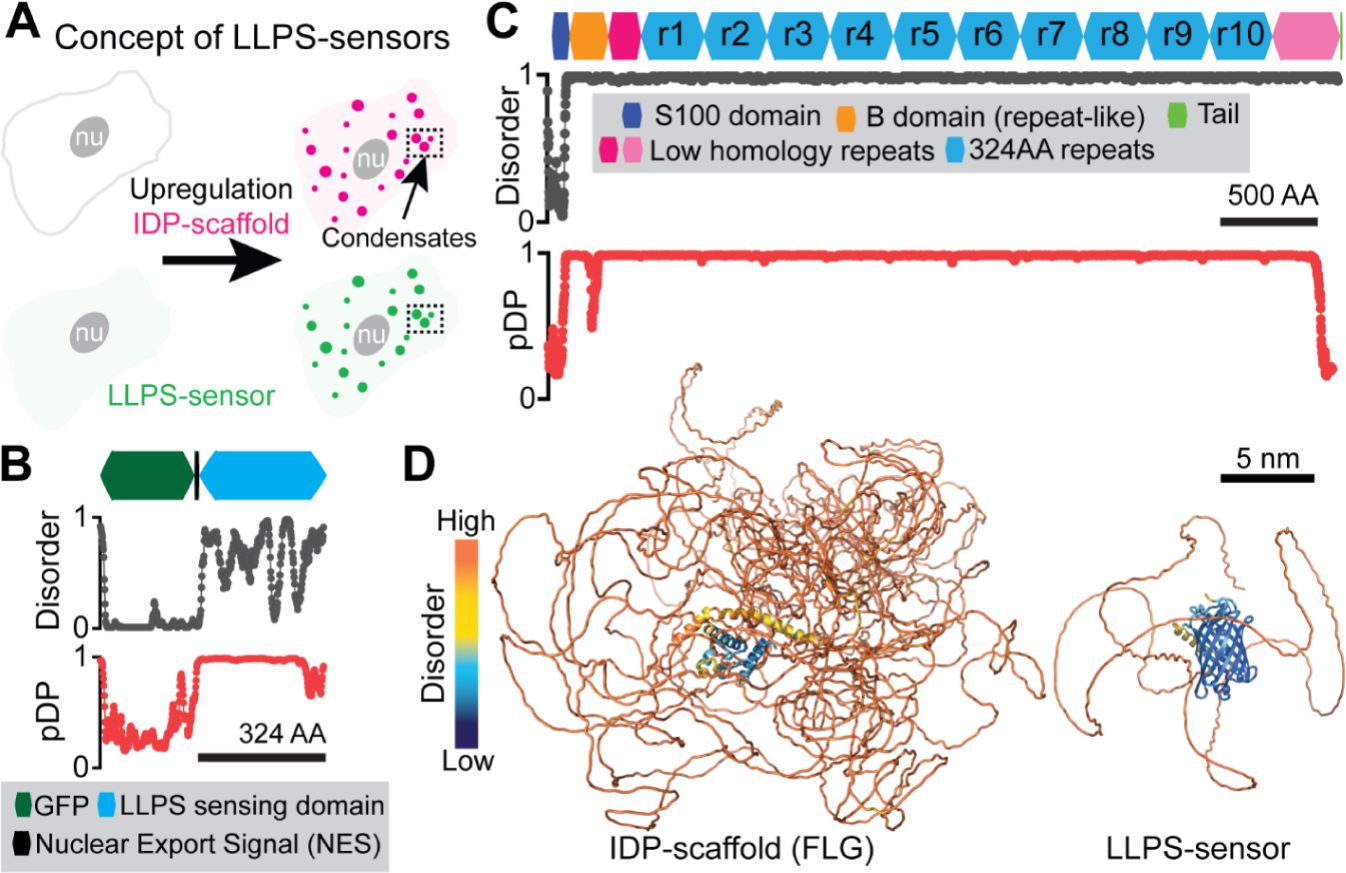
Concept and molecular features of genetically-encoded LLPS-sensors. (A) Exploiting IDP-encoded LLPS information from a target IDP-scaffold, a fluorescent LLPS-sensor switches from a diffuse (dim green; left) state to a condensate-enriched (bright; right) state as intracellular upregulation of an IDP-scaffold results in LLPS-driven assembly of biomolecular condensates (magenta). Without artifacts from molecular-level fluorescent tagging of IDP-scaffolds, live-cell imaging of LLPS-sensor fluorescence reveals the intracellular LLPS behavior of (untagged, nonfluorescent) IDP-scaffolds and their condensates. (B) Domain architecture, and corresponding disorder (where 1 = disorder) and droplet-promoting propensity (pDP; where 1 indicates highest LLPS-information content) plots for a recently developed LLPS-sensor (Sensor A) deployed to probe cytoplasmic epidermal condensates in mice.^7^ (C) Domain architecture, and corresponding disorder and droplet-promoting propensity plots for FLG, the His-rich IDP-scaffold of human epidermal condensates. (D) AlphaFold2 predictions of the molecular 3D structure and disorder for FLG and Sensor A.

The concept of LLPS-sensors involves a shift in focus from subcellular tracking of IDP-scaffolds to live-cell observations that report on the assembly and liquid-dynamics of their condensates. This shift eliminates the need to directly label the IDP-scaffold, creating the opportunity to engineer a protein that is sensitive to the underlying density phase transition (Figure 1A,B). We hypothesized that an engineered IDP that shares LLPS-relevant information (e.g., sequences responsible for charge–charge, cation–pi, pi–pi, hydrogen bonding, and hydrophobic interactions) with an IDP-scaffold can be evolved to engage in weak but multivalent interactions that only become substantial when the IDP-scaffold resides within dense condensates. If fused to a fluorescent protein, the resulting LLPS-sensor would experience a dramatic gain in signal-to-noise ratio as it accumulates within nascent condensates (Figure 1A).

We previously reported that Sensor A (Figure 1B), an engineered IDP that incorporates the LLPS-relevant information of FLG (Figure 1C), successfully exposed FLG-driven intracellular LLPS dynamics in the skin of genetically engineered mice. We also showed that Sensor A could probe untagged endogenous epidermal condensates in mouse and human skin, despite mouse FLG and human FLG lacking sequence conservation. Sensor A is comprised of two domains: (i) an engineered IDP with little sequence identity to FLG repeats, and (2) an engineered green fluorescent protein (Figure 1B,D). FLG exhibits high disorder and LLPS-relevant information across nearly the entirety of its repetitive architecture (Figure 1C,D). Here, we take advantage of this repetitive architecture to generate FLG-like (r8)_n_ proteins with variable (n) copies of the domain r8 (Figure S1A,B), creating a new testbed of IDP-scaffolds to quantitatively study the evolution and performance of epidermal LLPS-sensors.

To examine the tunability of epidermal LLPS-sensors, we considered mRFP1-(r8)_8_-Ctail scaffolds (Figure S1A) and their condensates as mimics of FLG and their KGs. In the case of FLG and notably mRFP1-(r8)_8_-Ctail, a single repeat domain (r8) contains all the scaffold-specific LLPS information (Figures 1C and S1B). Importantly, we do not interpret the high droplet-promoting scores in Figure 1B,C as reflective of a high probability of intracellular phase separation. We treat them as a metric of LLPS-information content, since we previously showed that (r8)_1_ and (r8)_2_ do not drive intracellular condensate formation even at extremely high expression levels.^7^ Sensor A, as a reference, uses an IDP-sensing domain (ir8H2) that shares little sequence identity to r8 (21.4%) but preserves the overall amino acid composition of r8^7^ and hence its high droplet-promoting scores (Figures 1B and S1B).

**Figure 2:**
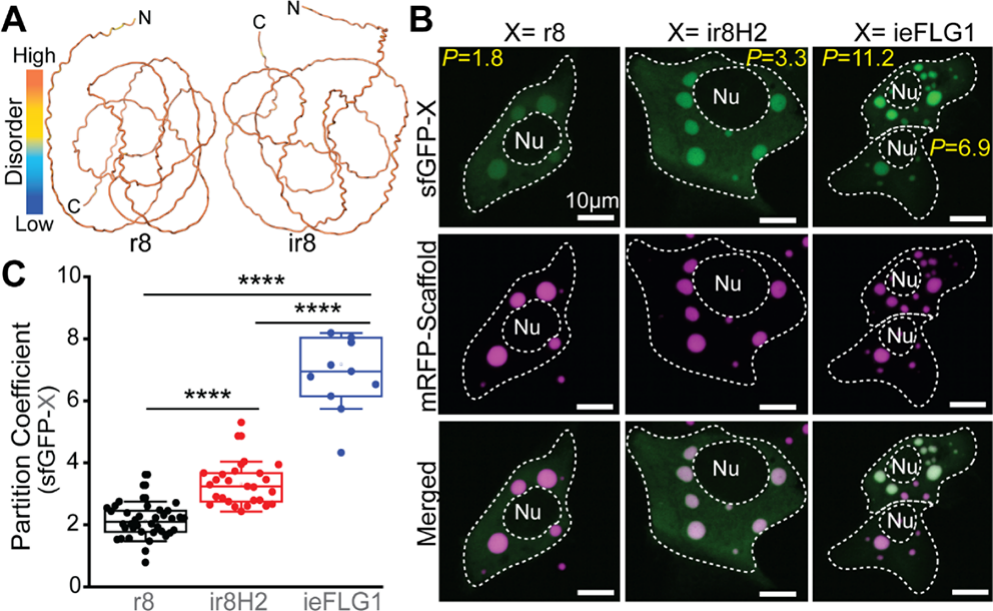
Sequence heuristics to encode LLPS in IDPs guide the evolution of tunable LLPS-sensing domains. (A) AlphaFold2 prediction of the 3D structure and disorder for a native FLG repeat domain (r8; Figure 1C) and a dissimilar domain (ir8) generated by reversing the sequence of r8 —willfully writing the r8 sequence from C-terminus to N-terminus. This sequence reversal destroys sequence similarity, while conserving intrinsic disorder and overall amino acid composition, which helps preserve key LLPS information in IDPs. The r8 domain encodes LLPS-relevant information (Figure S1A,B) but does not drive intracellular LLPS.^7^ (B,C) Live-cell images (B) and quantifications (C) of LLPS-sensor variants partitioning into mRFP1-(r8)_8_-Ctail condensates. The variants share a fixed fluorescent protein domain (sfGFP) but differ in the LLPS-sensing domain (X). ir8H2: variant of ir8 with H > Y mutations. ieFLG1: a de novo engineered r8-like sequence enriched for LLPS-relevant features. *P*: Partition Coefficient. Nu: nucleus. Each data point corresponds to the LLPS-sensor partition coefficient across all sizable condensates in a cell. Asterisks denote statistical significance (*p* ≤ 0.0001).

We cotransfected genes encoding a library of LLPS-sensor designs (Table S1) and mRFP1-(r8)_8_-Ctail into immortalized human keratinocytes (HaCATs) under conditions where no endogenous FLG is expressed. We note that FLG is a marker of late epidermal differentiation and stratification. Focusing on the IDP-sensing domain, we considered r8 as the simplest IDP that shares LLPS-information with our scaffold (Figures 2A and S1C). We fused r8 to a nuclear export signal (LELLEDLTL)^45^ and to superfolder GFP (sfGFP; Table S2) with the same architecture as Sensor A in Figure 1B. Despite the r8 domain being shared between this putative LLPS-sensor and the target IDP-scaffold, this initial sensor design appeared predominantly diffuse throughout the cytosol with little sensor signal colocalized exclusively with mRFP1-(r8)_8_-Ctail condensates (Figure 2B). Quantifying their recruitment to condensates, we measured the partition coefficient (*P*), that is the ratio of LLPS-sensor signal present within condensates compared to the cytosol. The sfGFP-tagged r8 design exhibited a low average partition coefficient (Figure 2C; *p* = 2.13 ± 0.55). The poor performance of this primitive sensor design showcases the need to evolve scaffold-specific LLPS-sensors for sensitive marking of target condensates.

To optimize the IDP-sensing domain, we turned to IDPs with low sequence identity to r8. In principle, sustained intracellular expression of LLPS-sensors with high sequence identity to IDP-scaffolds risks disruption of functional scaffold-specific biomolecular interactions. We previously applied a simple but dramatic sequence modification to r8: inversion of the syntax by reading its sequence in the unorthodox C- to N-terminal direction. The resulting protein (ir8) minimized sequence identity to r8 (25.6%) but preserved its overall composition and disorder (Figure 2A). Unlike scrambling, this strategy keeps the relative spacing and patterning of residues that function as stickers and spacers, which are important sources of LLPS-information.^46−48^ We note, however, that sequence inversion of IDP domains can shift LLPS dynamics.^7,49,50^

Using the ir8 domain, we previously introduced 18 histidine (His) to tyrosine (Tyr) mutations that mapped to naturally occurring His > Tyr mutations in r8 and r9 of FLG—accounting for ∼50% of the 37 His residues in r8. These His > Tyr mutations are known to increase LLPS propensity.^51,52^ We previously named the resulting His-rich (5.9%) and Tyr-rich (6.5%) sequence as ir8H2, which corresponds to the IDP-sensing domain of Sensor A in Figure 1B. Fusing ir8H2 to sfGFP and comparing it with r8 for the first time, we saw a significant improvement in its ability to distinguish mRFP1-(r8)_8_-Ctail condensates from the cytosolic background signal (Figure 2B). Quantitatively, the average partition coefficient increased by 55% (Figure 2C; *p* = 3.3 ± 0.7). Departing from r8 and its variants, we then considered ieFLG1, a 200-residue IDP (Figure S1C) consisting of five repeats of a zwitterionic His-rich (15%) sequence (SYGRHGSDGHGARDSQEHYGQRQHSHGSRDGQYSHSGDRG) designed de novo to match the composition of r8 while augmenting LLPS-relevant features, namely high tyrosine (7.5%) content.^7^ ieFLG1 shares little sequence identity to r8 (20.1%) and to ir8H2 (19.8%). When fused to sfGFP, ieFLG1 prominently marked mRFP1-(r8)_8_-Ctail condensates (Figure 2B), outperforming r8 with a 3.4-fold increase in the average partition coefficient (Figure 2C; *p* = 7.2 ± 1.8). These observations demonstrated that the IDP-sensing domain can be evolved to modulate the sensitivity of LLPS-sensors.

**Figure 3:**
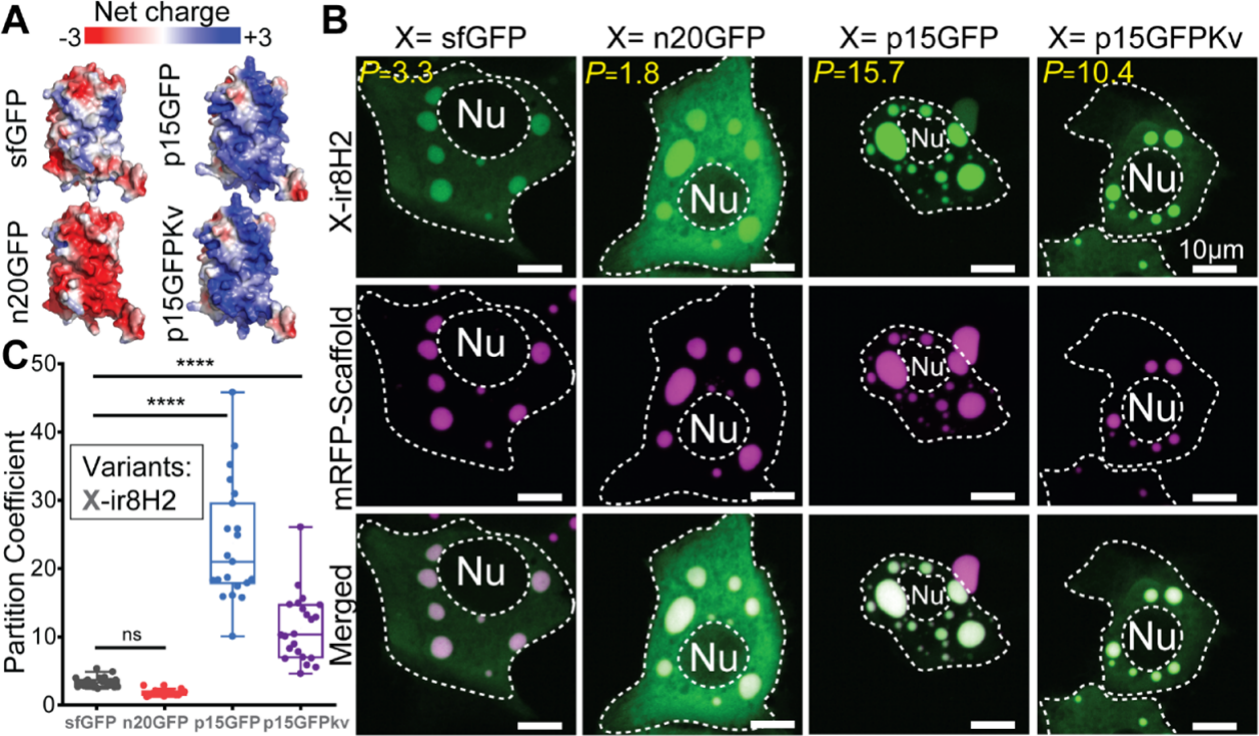
The fluorescent protein domain potently alters the sensitivity of the LLPS-sensing IDP domain. (A) AlphaFold2 predictions of the 3D surface of four variants of sfGFP, colored by net charge. (B,C) Live-cell images (B) and quantifications (C) of LLPS-sensor variants partitioning into mRFP1-(r8)_8_-Ctail condensates. The variants share a fixed LLPS-sensing IDP (ir8H2) but differ in the fluorescent protein domain (X). The sensitivity enhancement from p15GFP over all sfGFP variants, including p15GFPKv, was consistent across LLPS-sensing domains (see Figures S2 and S3). n20GFP: sfGFP with a negatively charged surface and −20 net charge. p15GFP: sfGFP with a positively charged surface and a +15 net charge. p15GFPKv: variant of p15GFP wherein surface-exposed Arg residues were mutated to Lys residues. *P*: Partition Coefficient. Nu: nucleus. Each data point corresponds to the LLPS-sensor partition coefficient across all sizable condensates in a cell. Asterisks denote statistical significance (*p* ≤ 0.0001). ns: not statistically significant (*p* > 0.05).

We next asked how the fluorescent protein domain of the LLPS-sensor dictates its sensitivity. We set out to test how the surface properties of closely related green fluorescent proteins (Figure 3A) influenced sensor performance. Specifically, we focused on surface charge as an accessible variable that is relevant to LLPS dynamics. sfGFP harbors a net charge of −6 and other groups have developed positive and negatively supercharged sfGFP variants.^53^ We selected two of these variants: n20GFP with a net charge of −20, and p15GFP with a net charge of +15. Fusing these sfGFP variants to our IDP-sensing domains, we found that n20GFP abolished the sensitive marking of mRFP1-(r8)_8_-Ctail by ir8H2 (Figure 3B) and ieFLG1 (Figure S2), yielding partition coefficients consistently lower than what we measured with sfGFP fusions (*p* = 1.8 ± 0.4; Figures 3C and S3). In contrast, p15GFP greatly improved overall recruitment of ir8H2 into mRFP1-(r8)_8_-Ctail condensates (Figure 3B), increasing the average partition coefficients 10-fold compared with sfGFP (*p* = 23.7 ± 8.9; Figure 3C). This enhanced sensitivity was also seen for p15GFP fusions to ieFLG1 (*p* = 35.5 ± 15.1; Figures S2 and S3) and to a lesser extent for the suboptimal r8 domain (*p* = 8.2 ± 2.5; Figure S3). Because the supercharging mutations in p15GFP favored LLPS-relevant Arginine (Arg) residues, we previously mutated the original eight Arg mutations into lysine (Lys or K).^7^ The resulting sfGFP variant, which we named p15GFPKv, retained the same net charge (Figure 3A) but lacked an Arg-decorated surface. Interestingly, p15GFPKv still outperformed sfGFP and allowed for sensitive marking of mRFP1-(r8)_8_-Ctail condensates with both ir8H2 (Figure 3B) and ieFLG1 (Figure S2), but with a consistent and significant 2-fold drop in the average partition coefficient compared with p15GFP fusions (*p* = 11.3 ± 5.0 for ir8H2 and *p* = 17.6 ± 7.1 for ieFLG1; Figures 3C and S3). These data demonstrated that the sensitivity of LLPS-sensors can be readily tuned by both the net charge and surface chemistry of the fluorescent protein domain. Our new data add to the growing evidence that fluorescent protein tags potently shift the LLPS dynamics of IDPs.^27^ Overall, our quantifications also showed for the first time that the two epidermal LLPS-sensors that we previously deployed in mice, Sensor A and Sensor B, feature optimized fluorescent protein (p15GFP) and IDP-sensing domains—with ir8H2 for Sensor A (Figure 1B) and ieFLG1 for Sensor B.^7^

### LLPS-Sensors Do Not Drive Intracellular Phase Separation

Despite carrying LLPS-relevant sequence information, optimized LLPS-sensors must remain soluble and diffuse in the absence of the IDP-scaffold or prior to the onset of intracellular LLPS (Figure 1A). Conceptually, LLPS-sensors should not exhibit concentration-dependent intracellular LLPS. In our extensive *in vivo* characterization of Sensor A, we previously saw that intracellular Sensor A in the spinous layer, prior to the onset of *flg* expression and KG assembly in the granular layer, appeared diffuse in the cytoplasm.^7^ Moreover, in mice that had undergone *flg* knockdown *in utero,* intracellular Sensor A signal appeared diffuse in all epidermal layers.^7^

**Figure 4:**
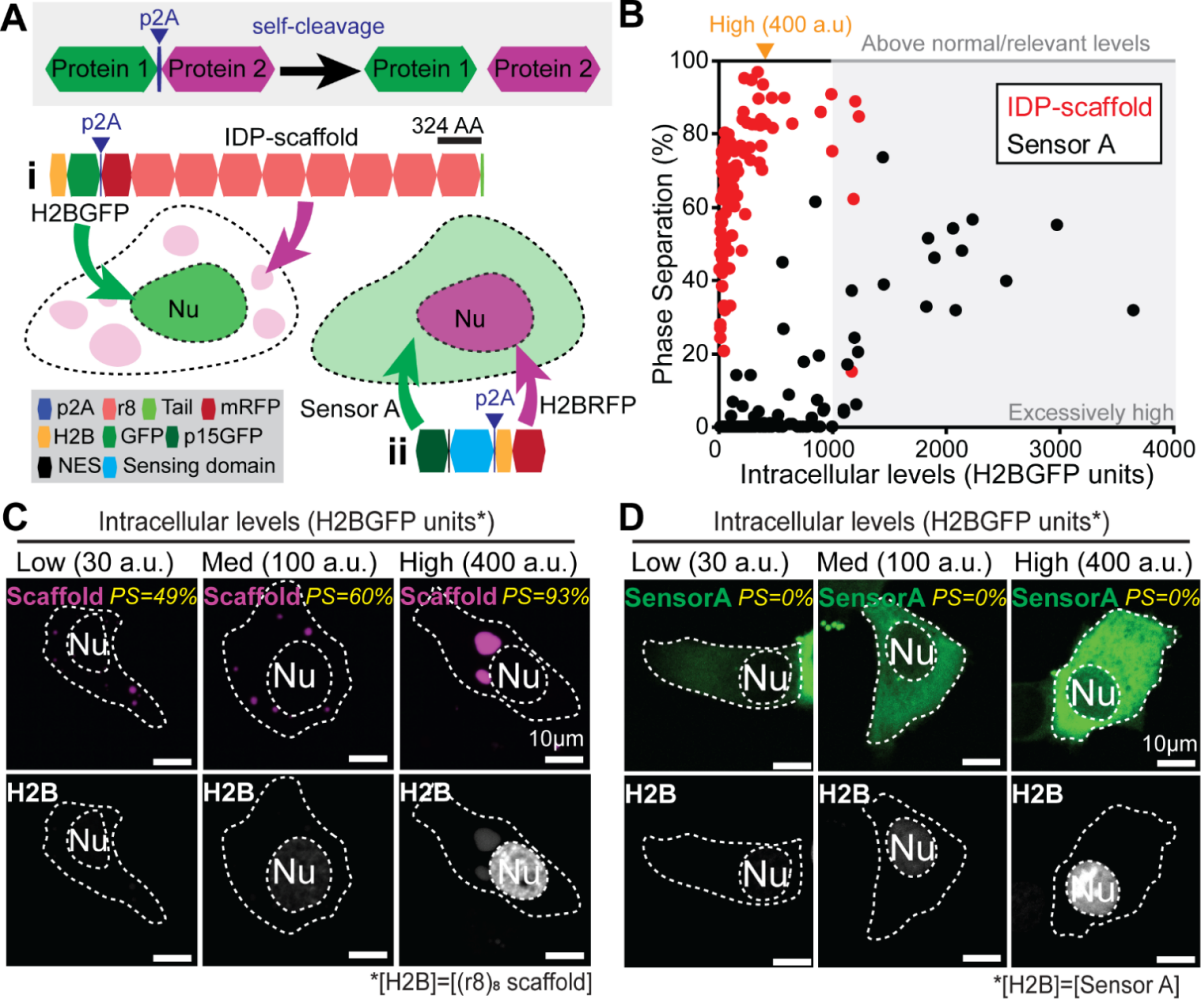
LLPS-sensors do not drive assembly of intracellular condensates. (A) Strategy to compare the concentration-dependent phase behavior of an FLG-like IDP-scaffold and its LLPS-sensor (Sensor A)—without coexpression of the scaffold. The P2A self-cleavage mechanism (light gray box) enables the use of fluorescent chromatin (H2B) as a consistent storage medium of the intracellular expression levels for both (i) mRFP1-(r8)_8_-Ctail and (ii) Sensor A. (B) Extent of intracellular LLPS for mRFP1-(r8)_8_-Ctail or Sensor A (alone) over a wide range of expression levels. Upon excessive overexpression (gray region), Sensor A aggregated without condensate formation (see Figure S4). H2BRFP units were converted to H2BGFP units using an experimentally validated (3:1) H2BGFP-to-H2BRFP ratio.^7^ (C) Live-cell images showing condensate formation by mRFP1-(r8)_8_-Ctail (magenta) over the entire range of protein expression levels in (B). (D) Live-cell images showing diffuse Sensor A signal (green) across an LLPS-relevant range of expression levels.

To further probe the intracellular behavior of Sensor A in the absence of an IDP-scaffold, we set out to study its intracellular localization as a function of expression levels in HaCATs, contrasting it with mRFP1-(r8)_8_-Ctail as a representative IDP-scaffold. To quantitatively compare the concentration-dependent behaviors of these divergent IDPs, we linked each IDP to a consistent live-cell reporter of intracellular concentration via equimolar coexpression of fluorescently tagged Histone H2B (H2B) (Figure 4A). In using nuclear chromatin to consistently store and report IDP concentration information, we sought to avoid quantification concerns related to drastic IDP size differences (303 kDa vs 64 kDa) and expected changes in localization (condensates vs diffuse). We used a self-cleavable P2A domain to generate equimolar amounts of these IDPs with either an mRFP1-tagged H2B protein (H2BRFP; linked to Sensor A) or a GFP-tagged H2B (H2BGFP; linked to the IDP-scaffold). Using live-cell imaging, we captured the intracellular distribution of each IDP and quantified the extent of phase separation in individual cells (Figure 4B), using the fluorescence intensity of the nuclear H2B protein as a consistent proxy for intracellular IDP concentration. To account for differences in fluorescent intensity between H2BRFP and H2BGFP, we transformed all H2BRFP measurements to H2BGFP units using our experimentally determined (3:1) H2BGFP-to-H2BRFP ratio.^7^

mRFP1-(r8)_8_-Ctail exhibited a sharp phase transition at low expression levels (Figure 4B), with the fraction of IDP-scaffold within liquid-like condensates approaching 100% at high expression levels (∼400 au;Figure 4C). Condensates were already prominent at low expression levels (∼30 au;Figure 4C). In contrast, Sensor A did not exhibit concentration-dependent LLPS over a wide concentration range (up to ∼1000 au,Figure 4B). For a handful of cells with abnormally high expression levels (>1000 au), mRFP1-(r8)_8_-Ctail signal within condensates dropped (Figure 4B) and Sensor A signal clustered into irregular aggregates (Figure S4) that lacked the liquid-like sphericity of the mRFP1-(r8)_8_-Ctail condensates. We considered this concentration regime as prone to artifacts from transfection. Within the LLPS-relevant range of concentrations, we confirmed that Sensor A at low (30 au), medium, (100 au) and high (400 au) intracellular levels distributed diffusely in the cytoplasm (Figure 4D). Together with our observations in Figure 3, these live-cell data demonstrated that optimized LLPS-sensors behave as soluble intracellular proteins that are highly sensitive to the density phase transitions exhibited by target IDP-scaffolds.

### Benchmarking LLPS-Sensor Performance with Ligand-Type Clients

Biomolecular condensates and their IDP-scaffolds interact with client biomolecules across a broad range of affinities. One prominent example involves ligand-type client proteins that bind one-to-one via a specific domain in the IDP-scaffold,^2,4,18,19,55^ We reasoned that fluorescent ligand-type client proteins offered an intriguing alternative to monitor the intracellular LLPS dynamics of target IDP-scaffolds with known binding domains.

**Figure 5:**
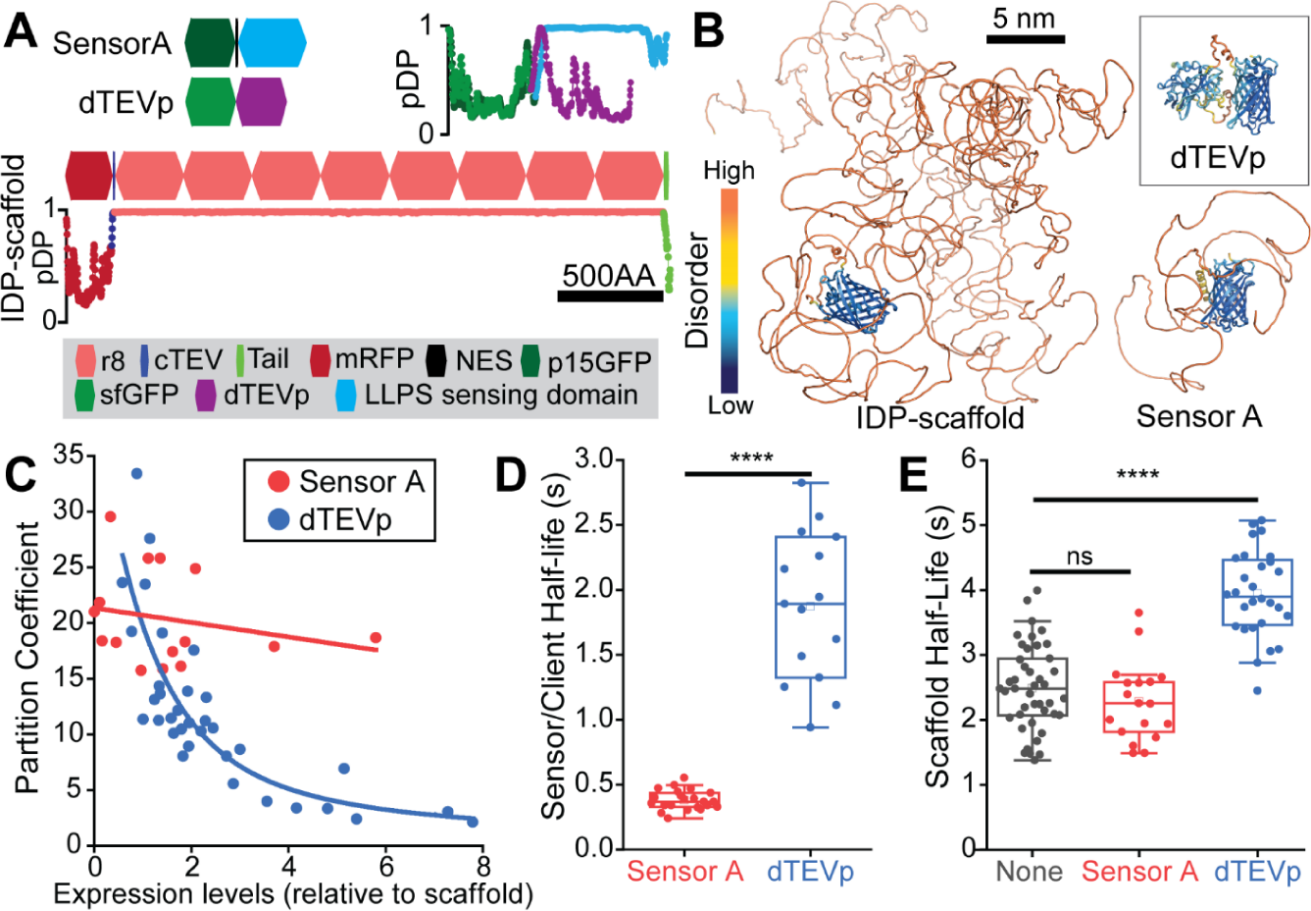
LLPS-sensors faithfully monitor condensate assembly and their liquid-like dynamics. (A) Domain architecture and droplet-promoting propensity (pDP; where 1 indicates highest LLPS information content) plots for three constructs: (i) mRFP1-cTEV-(r8)_8_-Ctail, an FLG-like IDP-scaffold engineered to incorporate a cTEV site; (ii) an engineered fluorescent client that binds to cTEV (dTEVp client); and (iii) Sensor A. dTEVp: dead variant of the Tobacco Etch Virus protease. cTEV: cleavage (and binding) site for TEVp. (B) AlphaFold2 predictions of the 3D structure and disorder for the three proteins in (A). (C) Quantification of Sensor A or dTEVp client partitioning into condensates as a function of their intracellular levels relative to the concentration of mRFP1-cTEV-(r8)_8_-Ctail. Unlike the dTEVp client, Sensor A sensitively marked condensates over a wide concentration range of sensor and IDP-scaffold levels. (D) Sensor A and dTEVp client recovery half-lives after internal photobleaching of the corresponding mRFP1-cTEV-(r8)_8_-Ctail condensates. Compared with the scaffold-interacting dTEVp client, Sensor A undergoes relatively unimpeded mixing within condensates. (E) IDP-scaffold recovery half-lives after internal photobleaching of the corresponding condensates with and without enrichment of Sensor A or dTEVp as indicated. The client–scaffold interactions slowed down the liquid-like dynamics of the scaffold (i.e., longer scaffold half-life), whereas Sensor A preserved the native liquid-like dynamics of the IDP-scaffold. Raw data in (D,E) reanalyzed from Supporting Information in ref (7). Asterisks: statistically significant (****p ≤ 0.0001). ns: not statistically significant (*p* > 0.05).

We set out to contrast the performance of Sensor A and a fluorescently tagged client for probing the intracellular LLPS dynamics of FLG-like IDP-scaffolds. We selected a dead variant of the Tobacco Etch Virus protease (dTEVp) fused to sfGFP as our model client (Figure 5A). We previously reported that sfGFP-dTEVp colocalizes with condensates assembled by mRFP1-cTEV-(r8)_8_-Ctail. This FLG-like IDP-scaffold features the short motif ENLYFQS, which is the known binding and cleavage site (cTEV) for TEVp. As suggested by the droplet-promoting propensity profiles in Figure 5A, this dTEVp client lacks sequence-level LLPS information. This is in line with our prior observation that sfGFP-dTEVp was excluded from condensates formed by mRFP1-(r8)_8_-Ctail modified with a defective mutant cTEV motif (ENLYFQR).^7^ While sfGFP-dTEVp is comparable in size to Sensor A, it lacks the disordered conformational dynamics of Sensor A and the target IDP-scaffold (Figures 5B and S5).

Fine control over the intracellular levels of genetically-encoded tools remains challenging, suggesting a requirement for intracellular LLPS-sensors to perform well over a wide concentration range. Here, we asked how the relative abundance of sensor/client to IDP-scaffold influenced the sensitive marking of intracellular condensates. We took advantage of the variable expression levels of mRFP1-cTEV-(r8)_8_-Ctail and either Sensor A or sfGFP-dTEVp upon transfection in HaCATs. At low intracellular levels, both Sensor A and sfGFP-dTEVp showed sensitive marking of mRFP1-cTEV-(r8)_8_-Ctail condensates with comparably high partition coefficients (*P* ∼ 20–30; Figure 5C). Sensor A sustained its high sensitivity even when expressed at higher levels than the IDP-scaffold (Figure 5C). In contrast, the partition coefficient of sfGFP-dTEVp decreased precipitously as the client-to-scaffold levels exceeded a ratio of 1 (Figure 5C), pointing to the concentration-dependent saturation of dTEVp-bound cTEV sites within condensates. We will consider the impact of the number of cTEV sites per IDP-scaffold later in this paper. These data suggested that ligand-type clients may have subpar performance in monitoring early intracellular LLPS dynamics dictated by the gain of IDP-scaffold expression—like *FLG* upregulation in the granular layer of human skin.

At late stages of intracellular LLPS, we previously reported that the liquid-like dynamics of mRFP1-cTEV-(r8)_8_-Ctail within condensates is sensitive to the binding of sfGFP-dTEVp but not Sensor A.^7^ We revisited these supplemental data and reanalyzed it to directly contrast the behavior and impact of Sensor A and sfGFP-dTEVp (Figure 5D,E). To do this, we considered internal fluorescence recovery after photobleaching (FRAP) measurements, this time as unnormalized recovery half-life values. These internal photobleaching experiments allowed us to quantify the relative diffusion dynamics of the IDP-scaffold and our fluorescently tagged tools within condensates. Sensor A showed greatly accelerated diffusion compared with sfGFP-dTEVp, exhibiting full recovery in less than a second (Figure 5D). sfGFP-dTEVp exhibited an average recovery half-life that was 4-fold longer than that of Sensor A (Figure 5D), with values comparable to the half-life of the IDP-scaffold (Figure 5E) despite the large size difference between client and scaffold (Figure 5B). These divergent dynamics reinforced the notion that Sensor A interacts very weakly with the IDP-scaffold, even within condensates. The surprisingly slow diffusion dynamics of dTEVp suggested substantial binding to the IDP-scaffold, which translated into a significant slowing down of IDP-scaffold dynamics (Figure 5E). On the other hand, prominent Sensor A recruitment to condensates did not significantly alter the IDP-scaffold half-life values, which were indistinguishable from control condensates (Figure 5E). Overall, our analyses showed that the ultraweak interactions between LLPS-sensor and IDP-scaffold allow for sensitive recruitment in a concentration-independent manner and without negative impact to IDP-scaffold liquid-like dynamics within condensates.

### LLPS-Sensors Innocuously Probe the Onset of Intracellular LLPS

**Figure 6:**
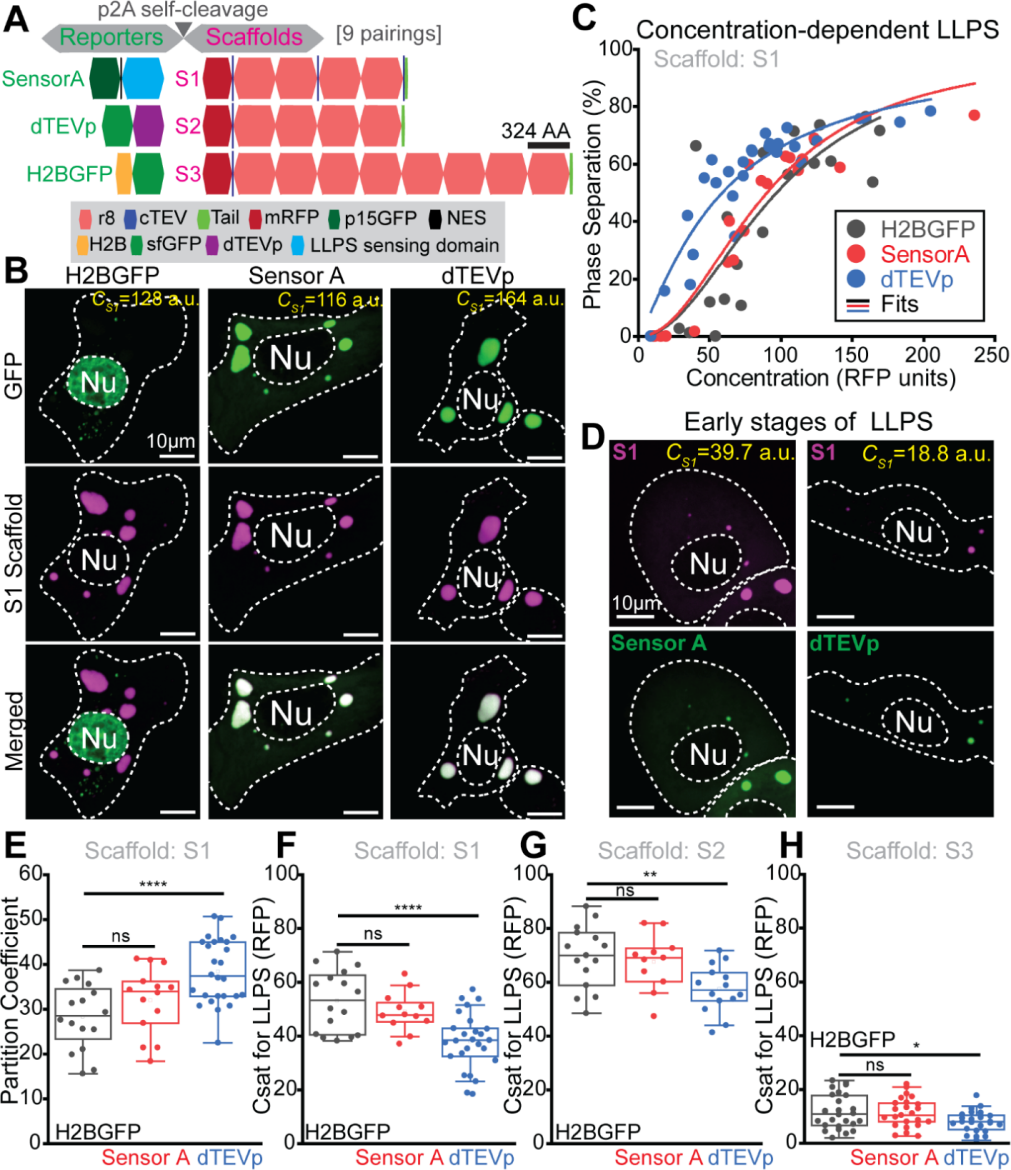
LLPS-sensors innocuously probe the intracellular phase separation dynamics of IDP-scaffolds. (A) Strategy to test the impact of Sensor A coexpression on the intracellular LLPS dynamics driven by three IDP-scaffolds (S1–S3). Self-cleavable p2A linkage ensures that Sensor A is abundantly present as the IDP-scaffold accumulates beyond its saturation concentration (*C*_sat_ for LLPS). This strategy was extended to a fluorescent ligand-type client (dTEVp) and a noninteracting control protein (nuclear H2BGFP), all p2A-linked to S1–S3. (B) Live-cell images of H2BGFP, Sensor A, and dTEVp client localization in cells with well-established S1 condensates and similar levels of S1 scaffold (∼120–160 RFP units). (C) Phase separation of the S1 scaffold as a function of its intracellular levels (RFP units) in cells with equivalent expression levels of H2BGFP (control), dTEVp client, or Sensor A. (D) Live-cell images of Sensor A or dTEVp recruitment to condensates in early LLPS. (E) Partition coefficients of S1 scaffold in the presence of H2BGFP (gray), Sensor A (red), and dTEVp (blue). (F–H) Critical concentrations for LLPS of S1 (F), S2 (G), and S3 (H) scaffolds in the presence of H2BGFP (gray), Sensor A (red), and dTEVp (blue). Asterisks denote statistical significance (* *p* < 0.05; **p ≤ 0.01; ****p ≤ 0.0001). ns: not statistically significant (*p* > 0.05).

Our initial observations focused on late-stage intracellular LLPS, which feature prominent biomolecular condensates that are amenable for FRAP assays. However, epidermal LLPS-sensors are expressed *in vivo* prior to the onset of LLPS. We turned our attention toward testing how Sensor A and sfGFP-dTEVp influenced the concentration-dependent onset of intracellular LLPS by a range of FLG-like IDP-scaffolds. To avoid confounding effects from variable intracellular ratios of IDP-scaffold to sensor/client, we deployed our P2A system to produce each IDP-scaffold and sensor/client at equimolar levels (Figure 6A). Specifically, across a wide range of IDP-scaffold levels, this equimolar strategy allowed us to test the impact of Sensor A in the challenging context of consistently coexisting with the IDP-scaffold at relatively high intracellular levels. Intuitively, findings from this regime should extend to relevant intracellular contexts wherein LLPS-sensors may be expressed at lower but variable levels compared with the native (untagged) IDP-scaffold. We also note that the equimolar system eliminates concerns from the variable occupancy of binding (cTEV) sites as a function of sfGFP-dTEVp levels—as we observed in Figure 5C.

Our IDP-scaffolds in Figure 6A varied in length (4 vs 8 repeats of r8) and the number of cTEV sites (1 vs 3). Using our live-cell imaging approach in HaCATs, we quantified the IDP-scaffold concentration outside (dilute phase) and within condensates for three IDP-scaffolds (S1–S3 in Figure 6A) in the presence of Sensor A, sfGFP-dTEVp or the nuclear-localized H2BGFP as a noninteracting control protein (Figure 6A). We treated the dilute phase concentration as a proxy for the *C*_sat_. Comparing the S1 and S2 scaffolds in the H2BGFP controls, we were surprised to notice that the two additional instances of the short cTEV motif in S1 (<1% of its residues) led to a significant drop in the *C*_sat_ and increased its partition coefficient (Figure S6A,B). Interestingly, when coexpressing with sfGFP-dTEVp, S1 condensates showed a nearly 4-fold increase in sfGFP-dTEVp recruitment compared with S2 condensates (Figure S6C). Sensor A partitioning was insensitive to the number of cTEV sites in these IDP-scaffolds, showing similar partition coefficients into condensates formed by S1 and S2 (Figure S6C). These data suggested that even large IDP-scaffolds may be sensitive to small sequence modifications required for engineered ligand-type clients.

Next, we examined whether Sensor A and sfGFP-dTEVp shifted the LLPS-relevant metrics of the S1 scaffold. Live-cell imaging showed robust Sensor A and dTEVp recruitment to large S1 condensates, while our control H2BGFP protein localized to the nucleus as expected (Figure 6B). Quantifying the concentration-dependent intracellular LLPS of S1, we saw that coexpression with dTEVp displayed a leftward shift in the extent of LLPS as a function of S1 levels when compared with H2BGFP (Figure 6C). In this system, the equimolar synthesis of S1 and dTEVp prevented saturation of the three cTEV sites in S1, resulting in sensitive marking of nascent condensates (Figure 6D). In line with the observed concentration-dependent shift, the *C*_sat_ for S1 in the presence of dTEVp dropped significantly while the scaffold partition coefficient increased compared with H2BGFP controls (Figure 6E,F). In notable contrast, Sensor A did not disrupt any of these key intracellular LLPS metrics for S1 (Figure 6C,E,F).

We repeated these analyses with the S2 scaffold, which is identical to S1 except that it features only one cTEV motif. We were motivated to test if limiting the client-scaffold interaction to one short motif rescues the client-linked disruptions of the endogenous LLPS dynamics. Sensor A and dTEVp sensitively marked S2 condensates (Figure S7A). We confirmed that Sensor A did not shift the concentration-dependent LLPS of S2 (Figure S7B), the *C*_sat_ (Figure 6G), and the partition coefficient (Figure S7C). dTEVp still noticeably shifted the concentration-dependent LLPS of S2 (Figure S7B), significantly reducing the *C*_sat_ (Figure 6G) and increasing the expected partition coefficient (Figure S7C). We then repeated these experiments with S3, which is similar to S2 but features double the number of r8 repeats (Figure 6A). For this large IDP-scaffold, Sensor A and dTEVp performed comparably, both faithfully reporting the LLPS dynamics and partition coefficients seen for the H2BGFP controls (Figure S8). Our sensitive measurements of *C*_sat_, however, still pointed to a small decrease in *C*_sat_ uniquely for S3 with dTEVp coexpression (Figure 6H). We also note that our prior observations on IDP-scaffold diffusion dynamics within condensates involved this S3 scaffold (Figure 5E), suggesting that large IDP-scaffolds remain susceptible to disruption within the protein-rich condensate environment. Overall, our new data demonstrated that our optimized LLPS-sensor consistently enabled high-fidelity intracellular probing of diverse IDP-scaffolds and that ligand-type clients may be suitable probes for a subset of IDP-scaffolds.

## Discussion

### Ultraweak Interactions for High-Fidelity Probing of Intracellular LLPS

**Figure 7:**
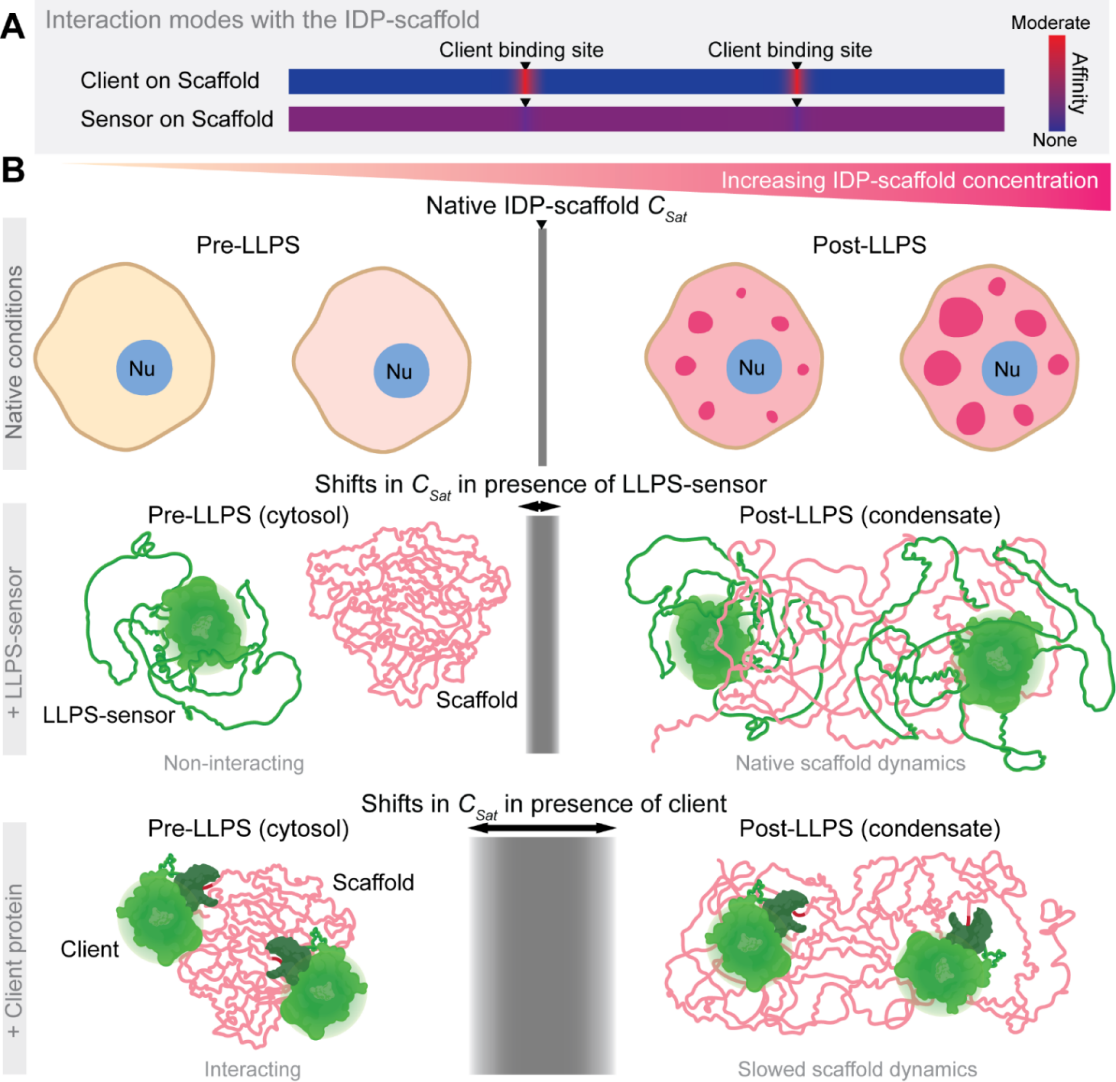
Ultraweak and phase-activated sensor–scaffold interactions enable sensitive and innocuous probing of intracellular condensate assembly and LLPS dynamics. (A) Ligand-type clients and LLPS-sensors differ in their interaction modes with IDP-scaffolds, distinguished by the affinity level and locations of the protein–protein interactions. (B) The resulting interaction dynamics help explain the unique ability of LLPS-sensors to faithfully monitor and report the native (top panel) LLPS dynamics of IDP-scaffolds. Prior to the saturation concentration (*C*_sat_) of the IDP-scaffold, LLPS-sensors (middle panel) do not interact with IDP-scaffolds that exist in a partially collapsed conformation reflective of favored intermolecular interactions,^54^ avoiding shifts to the native *C*_sat_ of the IDP-scaffold. Upon phase separation above *C*_sat_, IDP-scaffolds open to intermolecular interactions and weakly but multivalently engage with LLPS-sensors to sustain their LLPS-sensitive enrichment within nascent and established condensates—irrespective of the relative sensor levels. These phase-activated sensor–scaffold interactions are weak and highly dynamic, preventing shifts in the scaffold liquid-like dynamics within condensates. Unlike LLPS-sensors, coexpressed ligand-type clients (bottom panel) may bind to the IDP-scaffold with moderate affinity (i.e., even low μM level), allowing for interaction in the cytosol before the scaffold accumulates and phase separates above the native *C*_sat_. These substantial interactions can cause major shifts in the IDP-scaffold *C*_sat_, inhibiting or inducing (as we saw for the dTEVp client in Figure 6) intracellular phase separation, and inevitably slow down scaffold dynamics within assembled condensates.

Our findings for Sensor A and our dTEVp client suggest that the interaction mode with the IDP-scaffold is a key variable in the evolution of tools for high-fidelity intracellular probing of the LLPS dynamics of IDP-scaffolds and their condensates (Figure 7). Classical ligand-type clients bind to specific motifs along IDP-scaffolds with moderate-to-high affinity. Alternatively, LLPS-sensors engage in the same ultraweak intermolecular interactions that govern the LLPS of an IDP-scaffold (Figure 7A). This unique mode of interaction enables the sensor to engage weakly with the regions of the IDP-scaffold that encode LLPS-relevant information, with the bulk of transient scaffold-sensor interactions occurring within condensates. We demonstrate that these differences in interaction modes have critical implications for probing the concentration-dependent LLPS of the IDP-scaffold (Figure 7B).

Moderate-to-high affinity interactions between ligand-type clients and IDP-scaffolds may not discriminate the biophysical features of the IDP-scaffold before and after intracellular LLPS (Figure 7B). Rather, ligand-type clients that bind to the IDP-scaffold with high (1–100 nM) or moderate (>0.1–10 μM) affinity are likely to substantially interact with the IDP-scaffold in the dilute phase (Figure 7B). Our data demonstrate that these interactions can shift the underlying intracellular LLPS dynamics of select IDP-scaffolds. For large IDP-scaffolds that partially tolerate weak binding, like S3 in Figure S8, we showed that the *C*_sat_ (Figure 6H) and liquid-like dynamics within condensates remain sensitive to this binding (Figures 5E and 7B). In the case of our sfGFP-dTEVp client, its interactions with a range of IDP-scaffolds favored the assembly of dense condensates. Generally, we surmise that ligand-type clients may alter the solubility and conformational dynamics of IDP-scaffold chains to either increase or decrease intracellular LLPS propensity.^55,56^

LLPS-sensor interaction with the target IDP-scaffold is conditional upon the enrichment of IDP-scaffold within condensates, where they reside at high density and in an extended conformation (Figure 7B). Below the *C*_sat_ of the IDP-scaffold, the IDP chains are diffuse in the intracellular space at low concentrations that are unlikely to allow for substantial interaction with the LLPS-sensor—considering affinities in the 100 μM range for self-interacting IDPs. In this dilute regime, LLPS-prone IDP-scaffolds exist in a collapsed state dominated by intramolecular scaffold interactions, reducing the likelihood of heterotypic scaffold-sensor interactions.^54,57^ Without substantial interactions in the dilute phase, the biophysical and conformational properties of the IDP-scaffold are not perturbed prior to the onset of phase separation. Upon a phase transition, however, IDP-scaffold chains accumulate at high density within condensates—often well above 100 μM^7^—and adopt an extended conformation,^54,57^ promoting multivalent sensor–IDP–scaffold interactions that drive sensor accumulation within condensates. The interactions in the dense phase remain highly dynamic, preventing LLPS-sensors from interfering with the internal liquid-like dynamics of condensates.

Beyond our proof-of-principle work with epidermal LLPS-sensors, we acknowledge the challenge of engineering diverse LLPS-sensors for target IDP-scaffolds and their condensates. The evolution of engineered IDPs as bona fide LLPS-sensors demands a difficult balance between maximizing sensitive marking of target condensates while minimizing strong heterotypic interactions, which shift the LLPS dynamics of IDP-scaffolds in multicomponent condensates.^57,58^ Encouragingly, the engineering of IDP-sensing domains may accelerate with rapid progress in the sequence-level prediction of LLPS information in IDPs^8,59^ and in the computational prediction of their conformational and LLPS dynamics.^60−62^ These protein engineering efforts are poised to benefit from a growing understanding of the diverse LLPS grammars in IDP-scaffolds.^46−48,51,63^ The apparent specificity of IDP-driven weak interactions remains incompletely understood,^64^ which raises questions about the ability of specific LLPS-sensors to distinguish between dissimilar condensates. In keratinocytes, Sensor A without a nuclear export signal partitioned weakly into nucleoli, suggesting preferential marking of FLG condensates.^7^ However, LLPS-sensors fueled by ultraweak multivalent interactions may lack molecular information to distinguish condensates assembled by closely related IDP-scaffolds, such as RPTN (a FLG paralog) granules that appear to coexist with FLG condensates.^6^ In specific intracellular contexts, this crosstalk may facilitate comparative analyses of related condensates that share a particular LLPS-sensor.

### Outlook

We demonstrated that genetically-encoded LLPS-sensors can enable the high-fidelity biophysical probing of IDP-driven biomolecular condensates. Building on our studies of epidermal LLPS-sensors in mice, our new data convincingly show the potential to evolve biocompatible sensors with four key features: (1) highly tunable sensitivity, (2) concentration-independent partitioning into target biomolecular condensates, (3) faithful monitoring of the underlying concentration-dependent LLPS dynamics, and (4) innocuous probing of the liquid-like dynamics within condensates. While we focused on tools for biophysical probing, we note that LLPS-sensors can feature additional functional domains, such as enzymes that enable biomolecular characterization of condensates via proximity proteomics.^65^ LLPS-sensors fused to other proteins of interest may functionalize and drug endogenous condensates for therapeutic outcomes. In our efforts to evaluate sensor performance, we exposed the challenge of engineering ligand-type clients for probing the intracellular LLPS of IDP-scaffolds with high fidelity. We expect that the simplicity and generalizability of this approach will fuel interest in evolving novel ligand-type clients toward achieving minimal disruption of the endogenous LLPS dynamics. Given the enigmatic involvement of diverse IDP-scaffolds in physiological and disease mechanisms, we propose that an array of advanced biomolecular tools will be needed to rigorously and innocuously probe native and engineered condensates. We hope that our findings and conceptual progress will stimulate innovations that revolutionize the live-cell probing of IDP-scaffolds and their assemblies.

## Data Availability

All data needed to evaluate the conclusions in the paper are present in the paper and/or the Supplementary Information. The plasmids encoding relevant protein constructs can be provided by F.G.Q. pending scientific review and a completed material transfer agreement. Requests for these materials should be submitted to felipe.quiroz@emory.edu. Additional data related to this work may be requested from the authors.

## Supporting Information Available

Extended data of disorder plots, live-cell images, AlphaFold2 predictions, LLPS metrics of LLPS-sensor variants and dTEVp (Figures S1–S8); construct sequence information (Tables S1–S4) (PDF)

## Abbreviations

IDP: intrinsically disordered protein
LLPS: liquid–liquid phase separation
*C*_sat_: saturation concentration
KGs: keratohyalin granules
HaCATs: immortalized human keratinocytes
sfGFP: superfolder GFP
cTEV: TEVp cleavage site
dTEVp: dead Tobacco Etch Virus protease
FRAP: fluorescence recovery after photobleaching

## References

[ref1] Boeynaems S.; Alberti S.; Fawzi N. L.; Mittag T.; Polymenidou M.; Rousseau F.; Schymkowitz J.; Shorter J.; Wolozin B.; Van Den Bosch L. Protein Phase Separation: A New Phase in Cell Biology. Trends Cell Biol. 2018, 28 (6), 420–435. 10.1016/j.tcb.2018.02.004. 29602697 PMC6034118

[ref2] Banani S. F.; Lee H. O.; Hyman A. A.; Rosen M. K. Biomolecular condensates: Organizers of cellular biochemistry. Nat. Rev. Mol. Cell Biol. 2017, 18 (5), 285–298. 10.1038/nrm.2017.7. 28225081 PMC7434221

[ref3] Bracha D.; Walls M. T.; Brangwynne C. P. Probing and engineering liquid-phase organelles. Nat. Biotechnol. 2019, 37 (12), 1435–1445. 10.1038/s41587-019-0341-6. 31792412

[ref4] Alberti S.; Gladfelter A.; Mittag T. Considerations and Challenges in Studying Liquid-Liquid Phase Separation and Biomolecular Condensates. Cell 2019, 176 (3), 419–434. 10.1016/j.cell.2018.12.035. 30682370 PMC6445271

[ref5] Shin Y.; Brangwynne C. P. Liquid phase condensation in cell physiology and disease. Science 2017, 357 (6357), eaaf4382 10.1126/science.aaf4382. 28935776

[ref6] Avecilla A. R. C.; Quiroz F. G. Cracking the Skin Barrier: Liquid-Liquid Phase Separation Shines under the Skin. JID Innov. 2021, 1 (3), 100036 10.1016/j.xjidi.2021.100036. 34909733 PMC8659386

[ref7] Quiroz F. G.; Fiore V. F.; Levorse J.; Polak L.; Wong E.; Pasolli H. A.; Fuchs E. Liquid-liquid phase separation drives skin barrier formation. Science 2020, 367 (6483), eaax9554 10.1126/science.aax9554. 32165560 PMC7258523

[ref8] Hardenberg M.; Horvath A.; Ambrus V.; Fuxreiter M.; Vendruscolo M. Widespread occurrence of the droplet state of proteins in the human proteome. Proc. Natl. Acad. Sci. 2020, 117, 33254–33262. 10.1073/pnas.2007670117. 33318217 PMC7777240

[ref9] Wright P. E.; Dyson H. J. Intrinsically disordered proteins in cellular signalling and regulation. Nat. Rev. Mol. Cell Biol. 2015, 16 (1), 18–29. 10.1038/nrm3920. 25531225 PMC4405151

[ref10] Nedelsky N. B.; Taylor J. P. Bridging biophysics and neurology: Aberrant phase transitions in neurodegenerative disease. Nat. Rev. Neurol. 2019, 15 (5), 272–286. 10.1038/s41582-019-0157-5. 30890779

[ref11] Patel A.; Lee H. O.; Jawerth L.; Maharana S.; Jahnel M.; Hein M. Y.; Stoynov S.; Mahamid J.; Saha S.; Franzmann T. M.; et al. A Liquid-to-Solid Phase Transition of the ALS Protein FUS Accelerated by Disease Mutation. Cell 2015, 162 (5), 1066–1077. 10.1016/j.cell.2015.07.047. 26317470

[ref12] Alberti S.; Dormann D. Liquid–Liquid Phase Separation in Disease. Annu. Rev. Genet. 2019, 53 (1), 171–194. 10.1146/annurev-genet-112618-043527. 31430179

[ref13] Bouchard J. J.; Otero J. H.; Scott D. C.; Szulc E.; Martin E. W.; Sabri N.; Granata D.; Marzahn M. R.; Lindorff-Larsen K.; Salvatella X.; et al. Cancer Mutations of the Tumor Suppressor SPOP Disrupt the Formation of Active, Phase-Separated Compartments. Mol. Cell 2018, 72 (1), 19–36.e8. 10.1016/j.molcel.2018.08.027. 30244836 PMC6179159

[ref14] Lu B.; Zou C.; Yang M.; He Y.; He J.; Zhang C.; Chen S.; Yu J.; Liu K. Y.; Cao Q. Pharmacological Inhibition of Core Regulatory Circuitry Liquid–liquid Phase Separation Suppresses Metastasis and Chemoresistance in Osteosarcoma. Adv. Sci. 2021, 8 (20), 2101895 10.1002/advs.202101895. PMC8529446 34432948

[ref15] Brangwynne C.; Tompa P.; Pappu R. Polymer physics of intracellular phase transitions. Nat. Phys. 2015, 11 (11), 899–904. 10.1038/nphys3532.

[ref16] Holehouse A. S.; Kragelund B. B. The molecular basis for cellular function of intrinsically disordered protein regions. Nat. Rev. Mol. Cell Biol. 2024, 25 (3), 187–211. 10.1038/s41580-023-00673-0. 37957331 PMC11459374

[ref17] Mittag T.; Pappu R. V. A conceptual framework for understanding phase separation and addressing open questions and challenges. Mol. Cell 2022, 82 (12), 2201–2214. 10.1016/j.molcel.2022.05.018. 35675815 PMC9233049

[ref18] Banani S. F.; Rice A. M.; Peeples W. B.; Lin Y.; Jain S.; Parker R.; Rosen M. K. Compositional Control of Phase-Separated Cellular Bodies. Cell 2016, 166 (3), 651–663. 10.1016/j.cell.2016.06.010. 27374333 PMC4967043

[ref19] Kilgore H. R.; Young R. A. Learning the chemical grammar of biomolecular condensates. Nat. Chem. Biol. 2022, 18, 1298 10.1038/s41589-022-01046-y. 35761089 PMC9691472

[ref20] Ditlev J. A.; Case L. B.; Rosen M. K. Who’s in and who’s out—compositional control of biomolecular condensates. J. Mol. Biol. 2018, 430 (23), 4666–4684. 10.1016/j.jmb.2018.08.003. 30099028 PMC6204295

[ref21] Sawyer I. A.; Sturgill D.; Dundr M. Membraneless nuclear organelles and the search for phases within phases. Wiley Interdiscip. Rev.: RNA 2019, 10 (2), e1514 10.1002/wrna.1514. 30362243

[ref22] Bah A.; Forman-Kay J. D. Modulation of Intrinsically Disordered Protein Function by Post-translational Modifications. J. Biol. Chem. 2016, 291 (13), 6696–6705. 10.1074/jbc.R115.695056. 26851279 PMC4807257

[ref23] Owen I.; Shewmaker F. The Role of Post-Translational Modifications in the Phase Transitions of Intrinsically Disordered Proteins. Int. J. Mol. Sci. 2019, 20 (21), 5501 10.3390/ijms20215501. 31694155 PMC6861982

[ref24] Yang P.; Mathieu C.; Kolaitis R.-M.; Zhang P.; Messing J.; Yurtsever U.; Yang Z.; Wu J.; Li Y.; Pan Q.; et al. G3BP1 Is a Tunable Switch that Triggers Phase Separation to Assemble Stress Granules. Cell 2020, 181 (2), 325–345.e28. 10.1016/j.cell.2020.03.046. 32302571 PMC7448383

[ref25] Markmiller S.; Soltanieh S.; Server K. L.; Mak R.; Jin W.; Fang M. Y.; Luo E.-C.; Krach F.; Yang D.; Sen A.; et al. Context-dependent and disease-specific diversity in protein interactions within stress granules. Cell 2018, 172 (3), 590–604.e13. 10.1016/j.cell.2017.12.032. 29373831 PMC5969999

[ref26] Price I. F.; Wagner J. A.; Pastore B.; Hertz H. L.; Tang W. C. elegans germ granules sculpt both germline and somatic RNAome. Nat. Commun. 2023, 14, 1 10.1038/s41467-023-41556-4. 37749091 PMC10520050

[ref27] Dorner K.; Gut M.; Overwijn D.; Cao F.; Siketanc M.; Heinrich S.; Beuret N.; Sharpe T.; Lindorff-Larsen K.; Maria H. Tag with Caution-How protein tagging influences the formation of condensates. bioRxiv, 2024. 10.1101/2024.10.04.616694

[ref28] Christensen T.; Hassouneh W.; Trabbic-Carlson K.; Chilkoti A. Predicting transition temperatures of elastin-like polypeptide fusion proteins. Biomacromolecules 2013, 14 (5), 1514–1519. 10.1021/bm400167h. 23565607 PMC3667497

[ref29] Trabbic-Carlson K.; Meyer D.; Liu L. A.; Piervincenzi R.; Nath N.; LaBean T.; Chilkoti A. Effect of protein fusion on the transition temperature of an environmentally responsive elastin-like polypeptide: A role for surface hydrophobicity?. Protein Eng., Des. Sel. 2004, 17 (1), 57–66. 10.1093/protein/gzh006. 14985538

[ref30] Moses D.; Guadalupe K.; Yu F.; Flores E.; Perez A. R.; McAnelly R.; Shamoon N. M.; Kaur G.; Cuevas-Zepeda E.; Merg A. D. Structural biases in disordered proteins are prevalent in the cell. Nat. Struct. Mol. Biol. 2024, 31 (2), 283–292. 10.1038/s41594-023-01148-8. 38177684 PMC10873198

[ref31] Kaniyappan S.; Tepper K.; Biernat J.; Chandupatla R. R.; Hübschmann S.; Irsen S.; Bicher S.; Klatt C.; Mandelkow E.-M.; Mandelkow E. FRET-based Tau seeding assay does not represent prion-like templated assembly of Tau filaments. Mol. Neurodegener. 2020, 15, 39 10.1186/s13024-020-00389-1. 32677995 PMC7364478

[ref32] Caputo A.; Liang Y.; Raabe T. D.; Lo A.; Horvath M.; Zhang B.; Brown H. J.; Stieber A.; Luk K. C. Snca-GFP knock-in mice reflect patterns of endogenous expression and pathological seeding. eNeuro 2020, 7 (4), ENEURO.0007-20.2020 10.1523/ENEURO.0007-20.2020. PMC7470929 32788297

[ref33] Ibrahim K. A.; Grußmayer K. S.; Riguet N.; Feletti L.; Lashuel H. A.; Radenovic A. Label-free identification of protein aggregates using deep learning. Nat. Commun. 2023, 14 (1), 7816 10.1038/s41467-023-43440-7. 38016971 PMC10684545

[ref34] Ansari A. M.; Ahmed A. K.; Matsangos A. E.; Lay F.; Born L. J.; Marti G.; Harmon J. W.; Sun Z. Cellular GFP toxicity and immunogenicity: Potential confounders in in vivo cell tracking experiments. Stem Cell Rev. Rep. 2016, 12, 553–559. 10.1007/s12015-016-9670-8. 27435468 PMC5050239

[ref35] Bresser K.; Dijkgraaf F. E.; Pritchard C. E. J.; Huijbers I.-J.; Song J.-Y.; Rohr J. C.; Scheeren F. A.; Schumacher T. N. A mouse model that is immunologically tolerant to reporter and modifier proteins. Commun. Biol. 2020, 3 (1), 273 10.1038/s42003-020-0979-0. 32472011 PMC7260180

[ref36] Dai Y.; You L.; Chilkoti A. Engineering synthetic biomolecular condensates. Nat. Rev. Bioeng. 2023, 1 (7), 466–480. 10.1038/s44222-023-00052-6. PMC10107566 37359769

[ref37] Garg A.; González-Foutel N. S.; Gielnik M. B.; Kjaergaard M. Design of functional intrinsically disordered proteins. Protein Eng., Des. Sel. 2024, 37, gzae004 10.1093/protein/gzae004. 38431892

[ref38] Xu Y.; Qiao H. A hypothesis: Linking phase separation to meiotic sex chromosome inactivation and sex-body formation. Front. Cell Dev. Biol. 2021, 9, 674203 10.3389/fcell.2021.674203. 34485277 PMC8415632

[ref39] Jones D. T.; Cozzetto D. DISOPRED3: Precise disordered region predictions with annotated protein-binding activity. Bioinformatics 2015, 31 (6), 857–863. 10.1093/bioinformatics/btu744. 25391399 PMC4380029

[ref40] Madeira F.; Madhusoodanan N.; Lee J.; Eusebi A.; Niewielska A.; Tivey A. R. N.; Lopez R.; Butcher S. The EMBL-EBI Job Dispatcher sequence analysis tools framework in 2024. Nucleic Acids Res. 2024, 52, W521–W525. 10.1093/nar/gkae241. 38597606 PMC11223882

[ref41] Jumper J.; Evans R.; Pritzel A.; Green T.; Figurnov M.; Ronneberger O.; Tunyasuvunakool K.; Bates R.; Žídek A.; Potapenko A.; et al. Highly accurate protein structure prediction with AlphaFold. Nature 2021, 596 (7873), 583–589. 10.1038/s41586-021-03819-2. 34265844 PMC8371605

[ref42] Mirdita M.; Schütze K.; Moriwaki Y.; Heo L.; Ovchinnikov S.; Steinegger M. ColabFold: Making protein folding accessible to all. Nat. Methods 2022, 19 (6), 679–682. 10.1038/s41592-022-01488-1. 35637307 PMC9184281

[ref43] Webb B.; Sali A. Comparative protein structure modeling using MODELLER. Curr. Protoc. Bioinf. 2016, 54 (1), 5–6. 10.1002/cpbi.3. PMC5031415 27322406

[ref44] Pettersen E. F.; Goddard T. D.; Huang C. C.; Couch G. S.; Greenblatt D. M.; Meng E. C.; Ferrin T. E. UCSF Chimera—a visualization system for exploratory research and analysis. J. Comput. Chem. 2004, 25 (13), 1605–1612. 10.1002/jcc.20084. 15264254

[ref45] Woerner A. C.; Frottin F.; Hornburg D.; Feng L. R.; Meissner F.; Patra M.; Tatzelt J.; Mann M.; Winklhofer K. F.; Hartl F. U. Cytoplasmic protein aggregates interfere with nucleocytoplasmic transport of protein and RNA. Science 2016, 351 (6269), 173–176. 10.1126/science.aad2033. 26634439

[ref46] Bremer A.; Farag M.; Borcherds W. M.; Peran I.; Martin E. W.; Pappu R. V.; Mittag T. Deciphering how naturally occurring sequence features impact the phase behaviours of disordered prion-like domains. Nat. Chem. 2022, 14 (2), 196–207. 10.1038/s41557-021-00840-w. 34931046 PMC8818026

[ref47] Martin E. W.; Holehouse A. S.; Peran I.; Farag M.; Incicco J. J.; Bremer A.; Grace C. R.; Soranno A.; Pappu R. V.; Mittag T. Valence and patterning of aromatic residues determine the phase behavior of prion-like domains. Science 2020, 367 (6478), 694–699. 10.1126/science.aaw8653. 32029630 PMC7297187

[ref48] Rekhi S.; Garcia C. G.; Barai M.; Rizuan A.; Schuster B. S.; Kiick K. L.; Mittal J. Expanding the molecular language of protein liquid–liquid phase separation. Nat. Chem. 2024, 16, 1113–1124. 10.1038/s41557-024-01489-x. 38553587 PMC11230844

[ref49] Quiroz F. G.; Li N. K.; Roberts S.; Weber P.; Dzuricky M.; Weitzhandler I.; Yingling Y. G.; Chilkoti A. Intrinsically disordered proteins access a range of hysteretic phase separation behaviors. Sci. Adv. 2019, 5 (10), eaax5177 10.1126/sciadv.aax5177. 31667345 PMC6799979

[ref50] Li N. K.; Roberts S.; Quiroz F. G.; Chilkoti A.; Yingling Y. G. Sequence Directionality Dramatically Affects LCST Behavior of Elastin-Like Polypeptides. Biomacromolecules 2018, 19 (7), 2496–2505. 10.1021/acs.biomac.8b00099. 29665334

[ref51] Quiroz F. G.; Chilkoti A. Sequence heuristics to encode phase behaviour in intrinsically disordered protein polymers. Nat. Mater. 2015, 14 (11), 1164–1171. 10.1038/nmat4418. 26390327 PMC4618764

[ref52] Dzuricky M.; Rogers B. A.; Shahid A.; Cremer P. S.; Chilkoti A. De novo engineering of intracellular condensates using artificial disordered proteins. Nat. Chem. 2020, 12 (9), 814–825. 10.1038/s41557-020-0511-7. 32747754 PMC8281385

[ref53] Lawrence M. S.; Phillips K. J.; Liu D. R. Supercharging Proteins Can Impart Unusual Resilience. J. Am. Chem. Soc. 2007, 129 (33), 10110–10112. 10.1021/ja071641y. 17665911 PMC2820565

[ref54] Farag M.; Cohen S. R.; Borcherds W. M.; Bremer A.; Mittag T.; Pappu R. V. Condensates formed by prion-like low-complexity domains have small-world network structures and interfaces defined by expanded conformations. Nat. Commun. 2022, 13 (1), 7722 10.1038/s41467-022-35370-7. 36513655 PMC9748015

[ref55] Ruff K. M.; Dar F.; Pappu R. V. Ligand effects on phase separation of multivalent macromolecules. Proc. Natl. Acad. Sci. U. S. A. 2021, 118 (10), e2017184118 10.1073/pnas.2017184118. 33653957 PMC7958451

[ref56] Seim I.; Posey A. E.; Snead W. T.; Stormo B. M.; Klotsa D.; Pappu R. V.; Gladfelter A. S. Dilute phase oligomerization can oppose phase separation and modulate material properties of a ribonucleoprotein condensate. Proc. Natl. Acad. Sci. U. S. A. 2022, 119 (13), e2120799119 10.1073/pnas.2120799119. 35333653 PMC9060498

[ref57] Farag M.; Borcherds W. M.; Bremer A.; Mittag T.; Pappu R. V. Phase separation of protein mixtures is driven by the interplay of homotypic and heterotypic interactions. Nat. Commun. 2023, 14 (1), 5527 10.1038/s41467-023-41274-x. 37684240 PMC10491635

[ref58] Riback J. A.; Zhu L.; Ferrolino M. C.; Tolbert M.; Mitrea D. M.; Sanders D. W.; Wei M.-T.; Kriwacki R. W.; Brangwynne C. P. Composition-dependent thermodynamics of intracellular phase separation. Nature 2020, 581 (7807), 209–214. 10.1038/s41586-020-2256-2. 32405004 PMC7733533

[ref59] Lotthammer J. M.; Ginell G. M.; Griffith D.; Emenecker R. J.; Holehouse A. S. Direct prediction of intrinsically disordered protein conformational properties from sequence. Nat. Methods 2024, 21 (3), 465–476. 10.1038/s41592-023-02159-5. 38297184 PMC10927563

[ref60] Tesei G.; Trolle A. I.; Jonsson N.; Betz J.; Knudsen F. E.; Pesce F.; Johansson K. E.; Lindorff-Larsen K. Conformational ensembles of the human intrinsically disordered proteome. Nature 2024, 626 (8000), 897–904. 10.1038/s41586-023-07004-5. 38297118

[ref61] Joseph J. A.; Reinhardt A.; Aguirre A.; Chew P. Y.; Russell K. O.; Espinosa J. R.; Garaizar A.; Collepardo-Guevara R. Physics-driven coarse-grained model for biomolecular phase separation with near-quantitative accuracy. Nat. Comput. Sci. 2021, 1 (11), 732–743. 10.1038/s43588-021-00155-3. 35795820 PMC7612994

[ref62] Devarajan D. S.; Wang J.; Szała-Mendyk B.; Rekhi S.; Nikoubashman A.; Kim Y. C.; Mittal J. Sequence-dependent material properties of biomolecular condensates and their relation to dilute phase conformations. Nat. Commun. 2024, 15, 1912 10.1101/2023.05.09.540038. 38429263 PMC10907393

[ref63] Giraldo-Castano M. C.; Littlejohn K. A.; Avecilla A. R. C.; Barrera-Villamizar N.; Quiroz F. G. Programmability and biomedical utility of intrinsically-disordered protein polymers. Adv. Drug Delivery Rev. 2024, 212, 115418 10.1016/j.addr.2024.115418. PMC11389844 39094909

[ref64] De La Cruz N.; Pradhan P.; Veettil R. T.; Conti B. A.; Oppikofer M.; Sabari B. R. Disorder-mediated interactions target proteins to specific condensates. Mol. Cell 2024, 84 (18), 3497–3512.e9. 10.1016/j.molcel.2024.08.017. 39232584 PMC11416317

[ref65] Quiroz F. G.; Fuchs E. Phase separation sensors and uses thereof. US 20,230,061,804 A1, 2021.

