# Supplementary material for: Genetically-Encoded Phase Separation Sensors Enable High-Fidelity Live-Cell Probing of Biomolecular Condensates
Source: ACS Sens. 2025 Feb 23;10(3):1857–69. doi: 10.1021/acssensors.4c02851 (PMC11959610; doi:10.1021/acssensors.4c02851)
Supplement: Supplementary file 1 — se4c02851_si_001.pdf [file se4c02851_si_001.pdf]

Supporting Information

Genetically-encoded phase separation sensors enable high-fidelity live-cell probing of biomolecular condensates

Authors: Alexa Regina Chua Avecilla[1], Jeremy Thomas[1], Felipe Garcia Quiroz[1*]

Affiliations
[1]Wallace H. Coulter Department of Biomedical Engineering, Georgia Institute of Technology and Emory University, Atlanta, GA 30322, USA

∗Email: felipe.quiroz@emory.edu

This PDF file includes:

Figs. S1 to S8
Tables S1 to S4

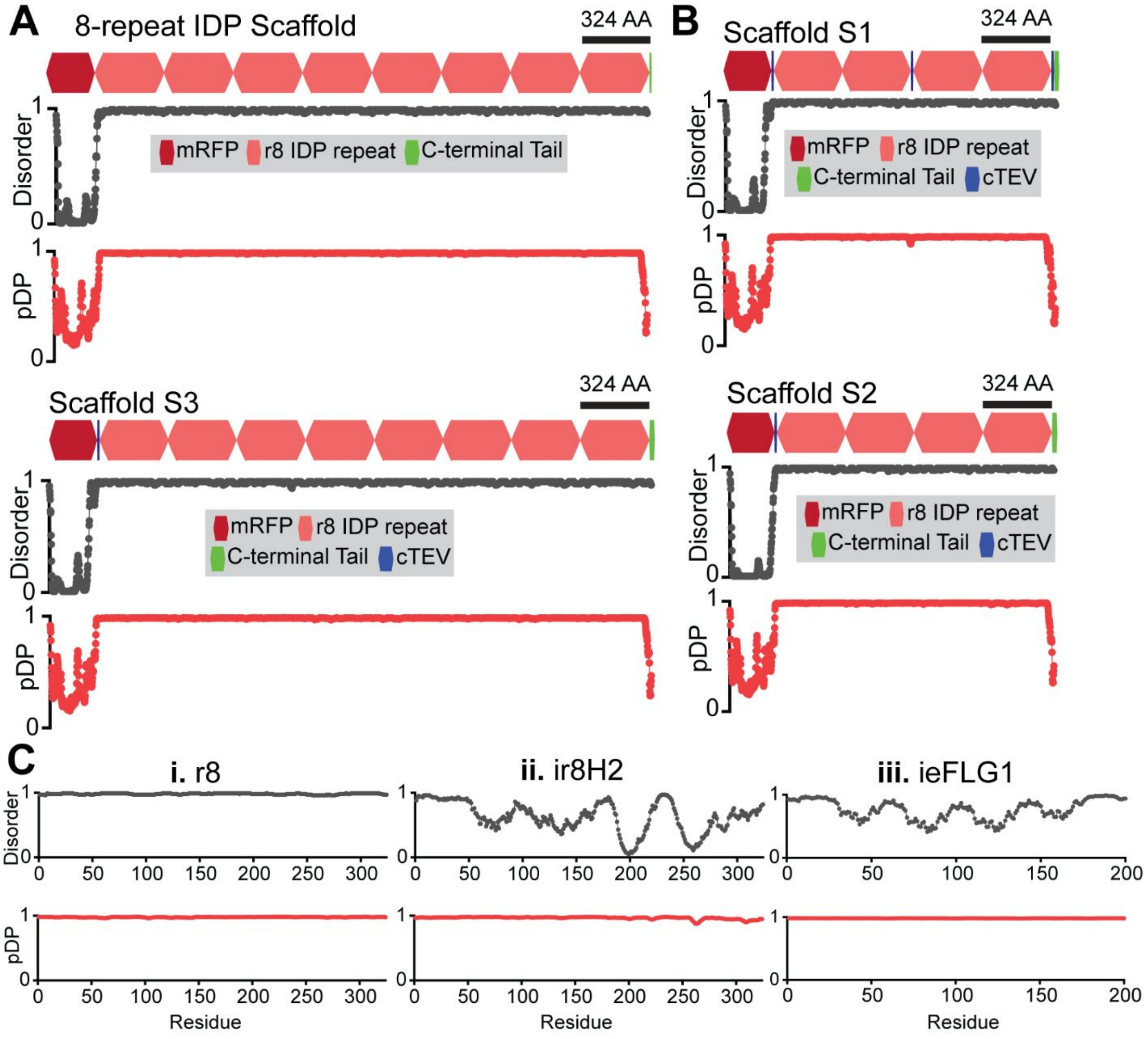

Fig. S1.

Architecture and features of FLG-like IDP-scaffolds and related LLPS-sensing IDP domains. (A-B) Domain architecture and corresponding disorder (where 1=disorder) and droplet-promoting propensity (pDP; where 1 indicates highest LLPS information content) plots for model IDP-scaffolds with **(A)** eight repeats of the r8 domain of human FLG, **(B)** four (S1 and S2) to eight (S3) repeats of r8 and one (S2-S3) to three (S1) cTEV sites. See Fig. 1C for FLG architecture. Note that r8 is ~95% identical to other repeat domains in FLG (e.g., r1-r7; r9-r10), hence the selection of a specific FLG repeat is inconsequential for the engineering of FLG-like IDP-scaffolds. **(C)** Disorder and pDP plots per residue for (i) r8 and related LLPS-sensing IDP domains (ii) ir8H2 and (iii) ieFLG1. Disorder prediction calculated with *DISOPRED3* and pDP calculated with FuzDrop.

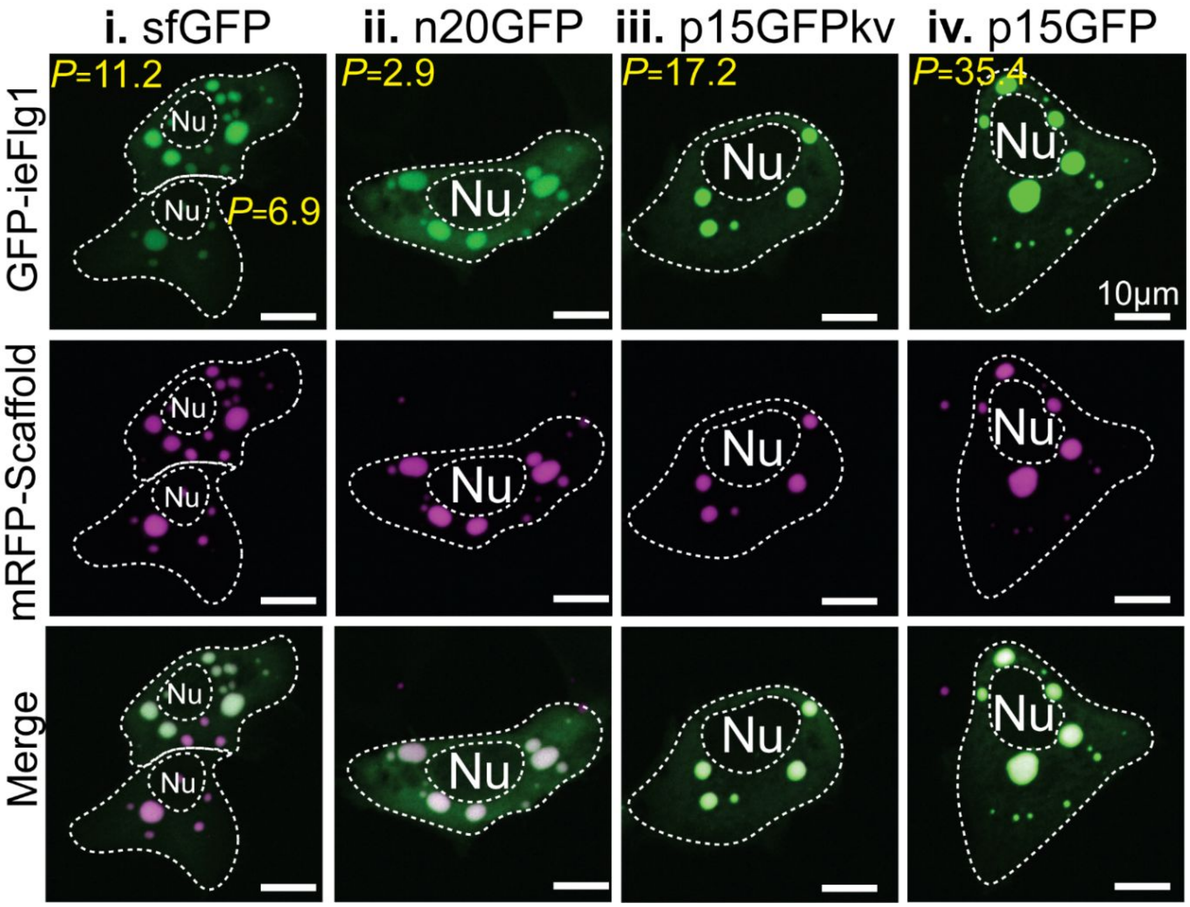

Fig. S2.

Live-cell images comparing the intracellular partitioning of ieFlg1-bearing LLPS-sensors based on the type of GFP-variant. Related to quantifications in Fig. S3 and in line with the observations for ir8H2-bearing LLPS-sensors in Fig. 3B-C and Fig. S3. mRFP-Scaffold: 8-repeat IDP scaffold as shown in Fig. S1A. *P* indicates the partition coefficient for the corresponding FP-ieFLG1 constructs in those specific cells.

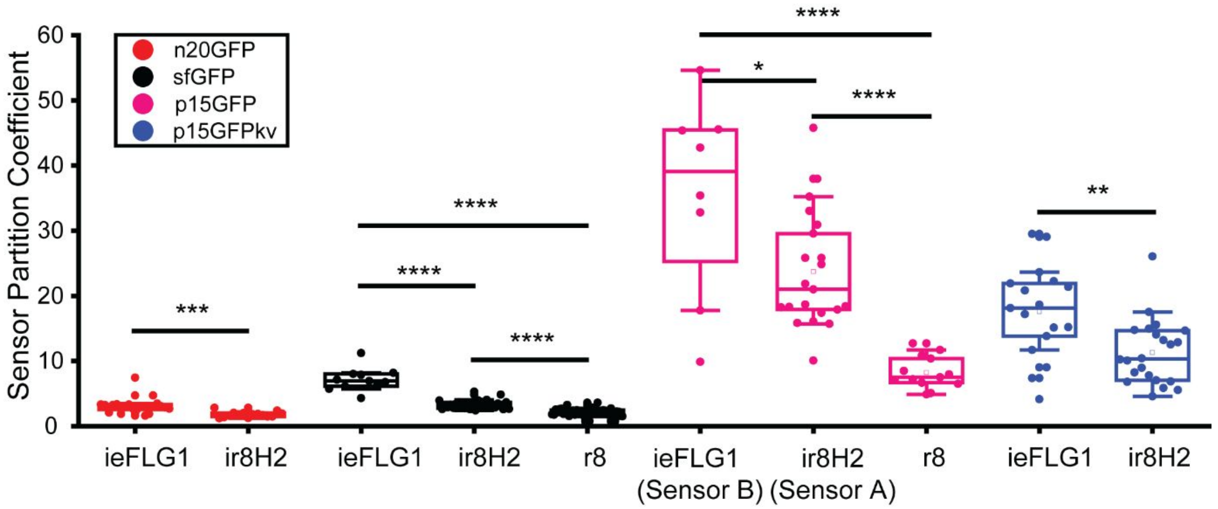

Fig. S3.

Tunable intracellular partition coefficient across LLPS-sensor designs. Quantitative analysis of the partition coefficient for all tested LLPS-sensor variants, varying IDP-sensor and fluorescent protein domains. These data correspond to sensor partitioning into intracellular condensates formed by the 8-repeat IDP-scaffold of Fig. S1A. These quantifications complement the images and data from Fig. 2, Fig. 3 and Fig. S2. Asterisks denote statistical significance (* = P≤0.05; ** = P≤0.01; *** = P≤0.001; **** = P≤0.0001).

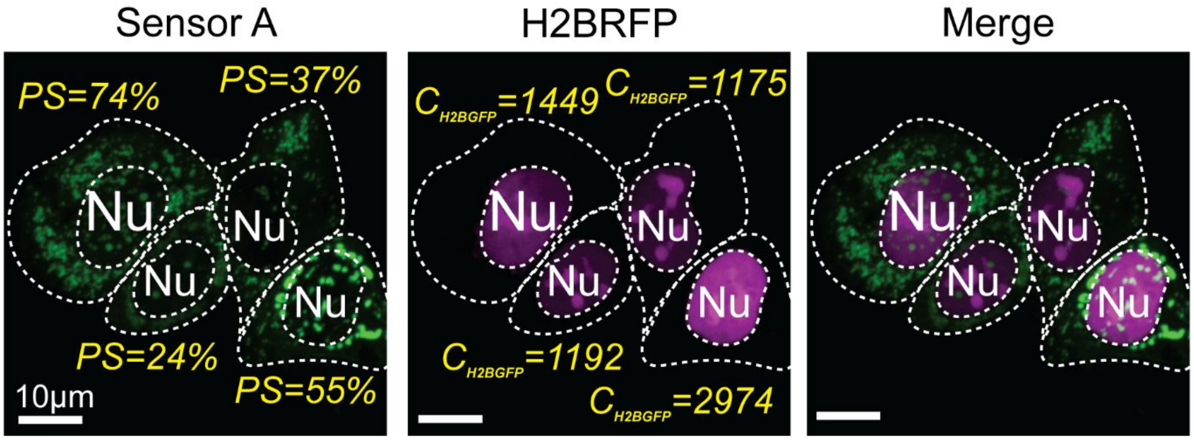

Fig. S4.

Excessively high intracellular levels of Sensor A results in aggregation rather than phase separation. Live-cell images with corresponding concentration (C; H2BGFP units) and percent "phase separation" (PS) measurements for cells expressing abnormally high levels of Sensor A. These levels are not usually achieved during our typical transfection conditions (e.g., when combining scaffold and sensor) and far exceed what we have previously reported in mice upon in utero transduction of developing keratinocytes with lentivirus [7]. Under these conditions, Sensor A signal revealed irregular clumps that differed morphologically from the liquid-like spherical condensates marked by Sensor A in the presence of (r8)n IDP-scaffolds (for n≥4), FLG (in primary human keratinocytes) and flg (in vivo, in mouse skin). While distinct from phase separation, we chose to quantify and report PS to capture the segregation of fluorescent signal that only occurs when Sensor A is expressed at these abnormally high levels. C is reported as CH2BGFP, converting CH2BRFP to CH2BGFP to account for our experimentally-determined 1:3 H2BGFP-to-H2BRFP ratio.

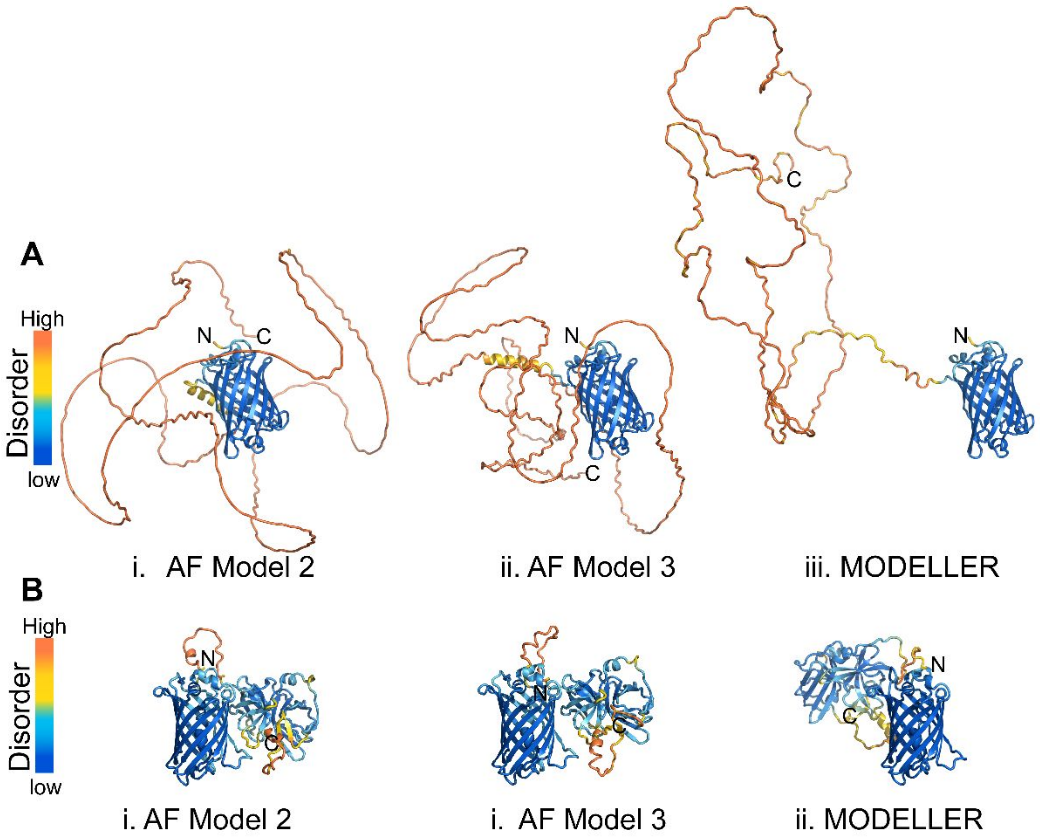

Fig. S5.

Distinct conformational dynamics between Sensor A and a ligand-type client. **(A-B)** Three snapshots (i-iii) of predicted 3D structures for (A) Sensor A and (B) our sfGFP-dTEVp client. **(A)** The IDP-sensor domain in Sensor A is predicted to sample widely divergent disordered conformations. In contrast, **(B)** the conformational dynamics of the two-domain client protein are largely restricted to small fluctuations in the disordered linker segment. Models (i)-(ii) were predicted by AlphaFold2 from complete protein sequences. Model (iii) was predicted through homology modeling with MODELLER from the separate predictions for each of the two domains in either the sensor or client.

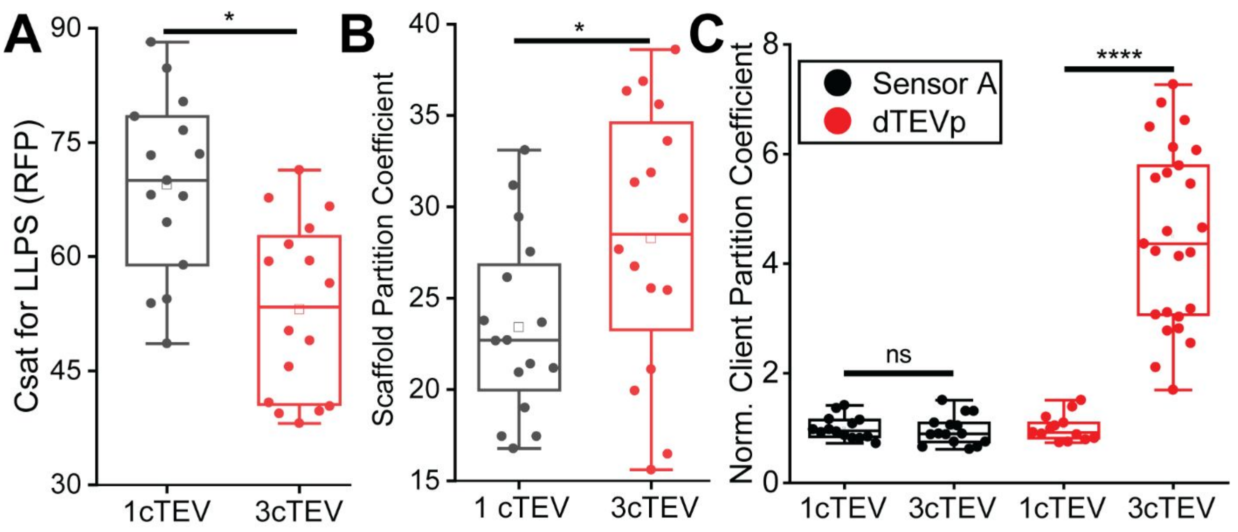

Fig. S6.

The number of client binding sites along a large IDP-scaffold alters its intracellular LLPS dynamics and client recruitment. **(A-B)** Scaffold critical concentration for LLPS (A) and partition coefficient (B) for 4-repeat r8 scaffolds that harbor either three (S1 in Fig. 6) or one (S2 in Fig 6) TEVp binding sites (cTEV) along their amino acid sequence. **(C)** Normalized partition coefficients for Sensor A or dTEVp in the presence of 4-repeat r8 scaffolds that harbor either one or three cTEV sites, using the mean partition coefficient of the corresponding 1cTEV constructs as reference for normalization. Asterisks denote statistical significance (* = P≤0.05; **** = P≤0.0001). ns: not statistically significant (p>0.05).

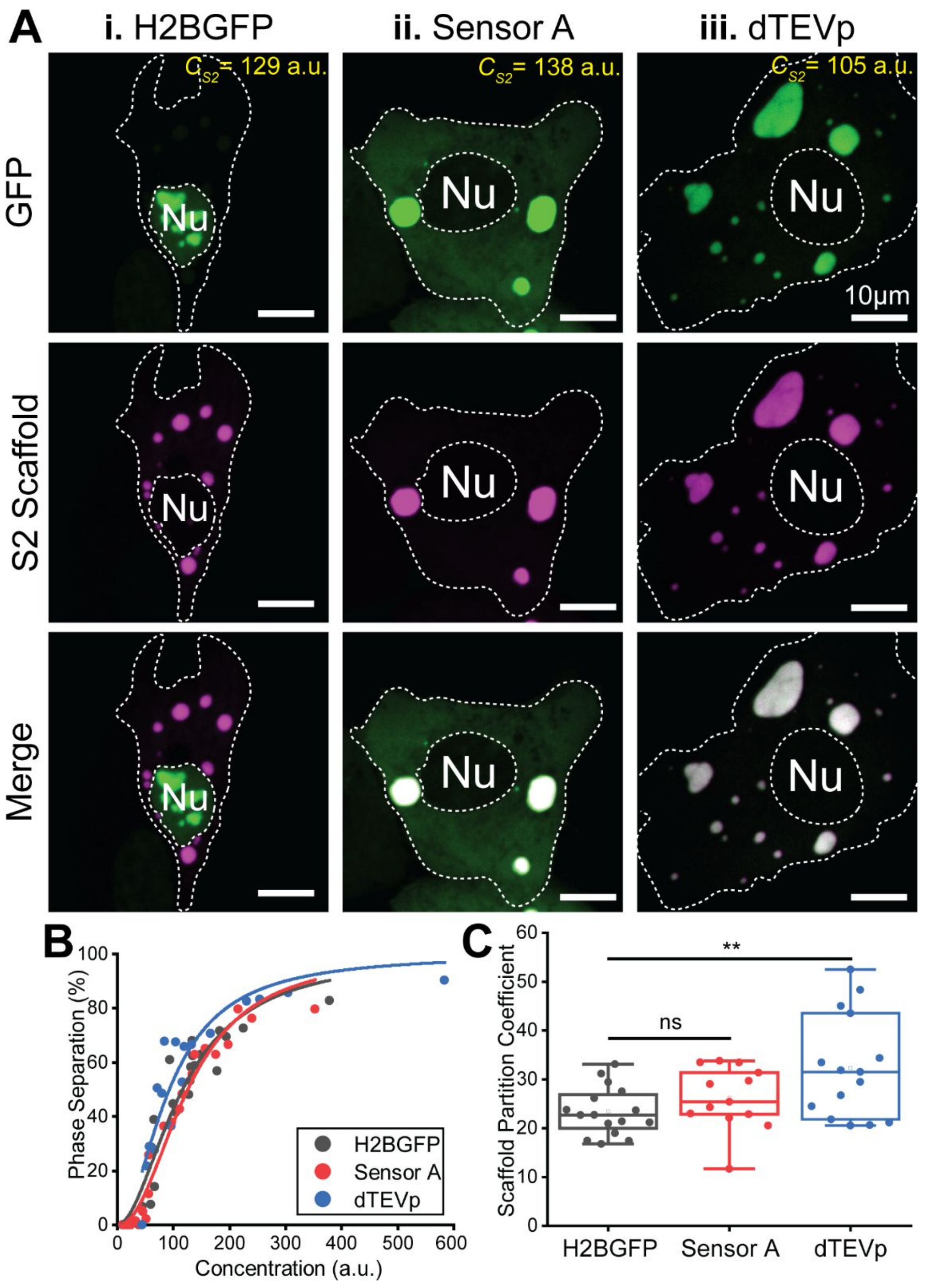

Fig. S7.

Intracellular LLPS behavior of large 4-repeat r8 scaffolds with one small binding site is sensitive to ligand-type client binding. (A) Live-cell images of (i) H2BGFP, (ii) Sensor A, (iii) dTEVp-client behavior in the presence of S2-scaffold (as in Fig. 6A) condensates. **(B)** Percent phase separation versus concentration of S2-scaffold in the presence of H2BGFP (grey), Sensor A (red), and dTEVp-client (blue). See Fig. 6G for quantifications of the dTEVp-induced shifts in the critical concentration for LLPS of the S2-scaffold. **(C)** Partition coefficients of S2-scaffold in

the presence of H2BGFP (grey), Sensor A (red), and dTEVp-client (blue). Asterisks denote statistical significance (** = $P \leq 0.01$). ns: not statistically significant ($p>0.05$).

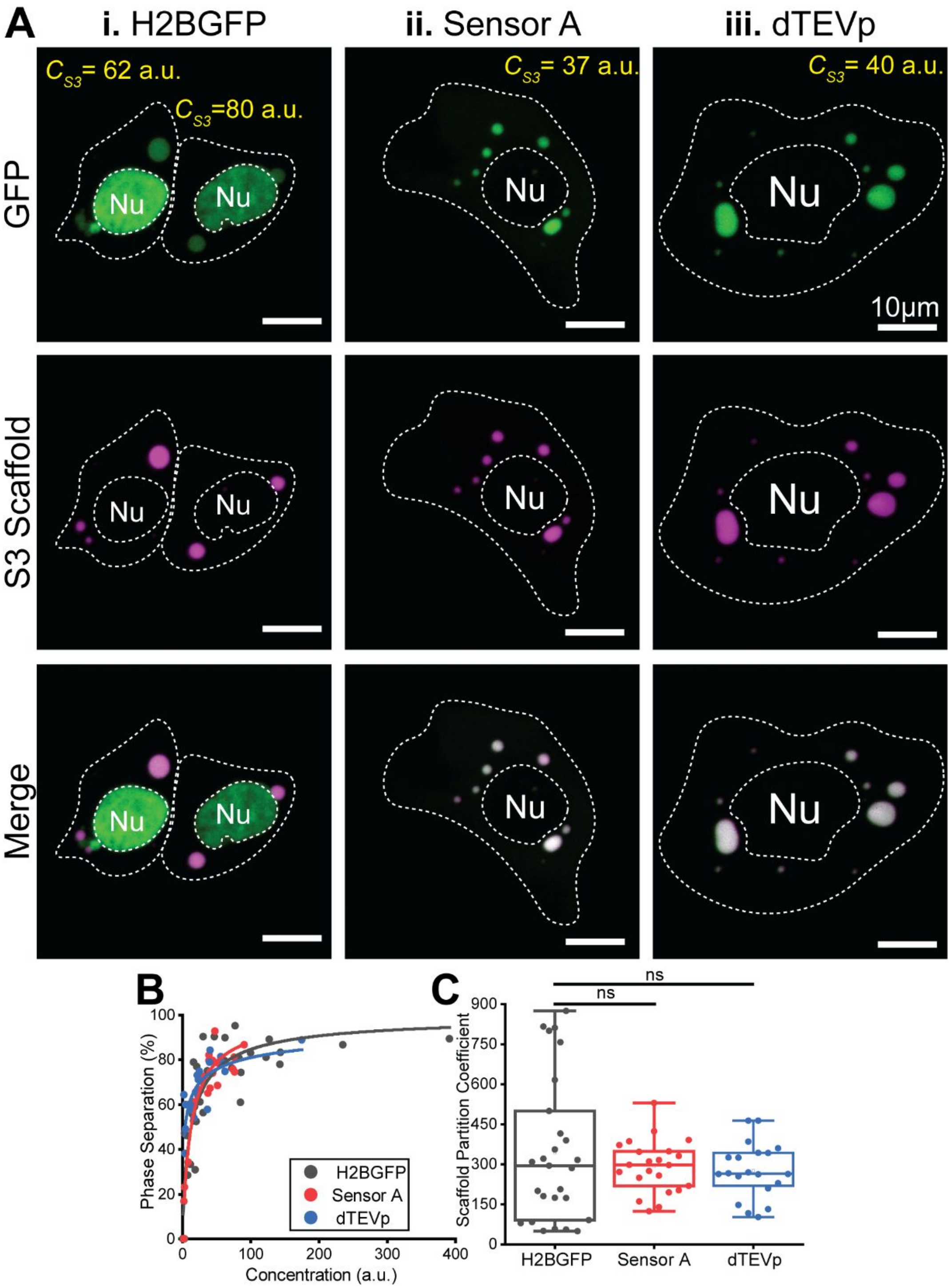

Fig. S8.

Intracellular LLPS behavior of very large 8-repeat r8 scaffolds is insensitive to ligand-type client binding. (A) Live-cell images of (i) H2BGFP, (ii) Sensor A, (iii) dTEVp-client behavior in the presence of S3-scaffold (as shown in Fig. 6A) condensates. **(B)** Percent phase separation versus concentration of S3-scaffold in the presence of H2BGFP (grey), Sensor A (red), and

dTEVp-client (blue). **(C)** Partition coefficients of S3-scaffold in the presence of H2BGFP (grey), Sensor A (red), and dTEVp-client (blue). ns: not statistically significant ($p>0.05$).

Table S1.
Sequence information for IDP-based sensing domains in Fig. 2 and Fig. S3.

Construct	Sequence
r8	QVSTHEQSESSHGWTGPSTRGRQGSRHEQAQDSSRHSASQDGQDTIRGHP<br>GSSRGGRQGYHHEHSVDSSGHSGSHHSHTTSQGRSDASRGQSGSRSASRTT<br>RNEEQSGDGSRHSGSRHHEASTHADISRHSQAVQGQSEGSRRSRRQGSSVS<br>QDSDSEGHSEDSERWSGSASRNHHGSAQEQLRDGSRHPRSHQEDRAGHGH<br>SADSSRQSGTRHTQTSSGGQAASSHEQARSSAGERHGSHHQQSADSSRHSG<br>IGHGQASSAVRDSGHRGYSGSQASDNEGHSEDSDTQSVSAHGQAGSHQQS<br>HQESARGRSGETSGHSGSFLY
ir8	YLFSGSHGSTEGSRGRASEQHSQQHSGAQGHASVSQTDSDESHGENDSAQ<br>SGSYGRHGSDRVASSAQGHGIGSHRSSDASQQHHSGHREGASSRAQEHSS<br>AAQGGSSTQTHRTGSQRSSDASHGHGARDEQHSRPHRSGDRLQEQASGHH<br>NRSASGSWRESDESHGESDSDQSVSSGQRRSRRSGESQGQVAQSHRSIDAH<br>TSAEHHRSGSHRSGDGSQEENRTTRSASRSGSQGRSADSRGQSTTHSHHSG<br>SHGSSDVSHEHHYGQRGGRSSGPHGRITDQGDQSASHRSSDQAQEHRSGQ<br>RGRTSPGTWGHSSESQEHTSVQ
ir8H2	YLFSGSHGSTEGSRGRASEQYSQQHSGAQGHASVSQTDSDESHGENDSAQ<br>SGSYGRHGSDRVASSAQGHGIGSHRSSDASQQYHSGYREGASSRAQEHSS<br>AAQGGSSTQTYRTGSQRSSDASYGHGARDEQHSRPYRSGDRLQEQASGHH<br>NRSASGSWRESDESHGESDSDQSVSSGQRRSRRSGESQGQVAQSYRSIDAH<br>TSAEYHRSGSYRSGDGSQEENRTTRSASRSGSQGRSADSRGQSTTHSHYSG<br>SYGSSDVSHEYYYGQRGGRSSGPYGRITDQGDQSASYRSSDQAQEYRSGQ<br>RGRTSPGTWGYSSESQEYTSVQ
ieFlg1	SYGRHGSDGHGARDSQEHYGQRQHSHGSRDGQYSHSGDRGSYGRHGSDG<br>HGARDSQEHYGQRQHSHGSRDGQYSHSGDRGSYGRHGSDGHGARDSQEH<br>YGQRQHSHGSRDGQYSHSGDRGSYGRHGSDGHGARDSQEHYGQRQHSH<br>GSRDGQYSHSGDRGSYGRHGSDGHGARDSQEHYGQRQHS<br>HGSRDGQYSHSGDRG

Table S2.
Sequence information for fluorescent proteins across sensor designs in Fig. 3.

Construct	Sequence
sfGFP	MGSKGEELFTGVVPILVELDGDVNGHKFSVRGEGEGDATNGKLTLKFICT TGKLPVPWPTLVTTLGYGVQCFSRYPDHMKRHDFFKSAMPEGYVQERTIS FKDDGTYKTRAEVKFEGDTLVNRIELKGIDFKEDGNILGHKLEYNFNSHN VYITADKQKNGIKANFKIRHNVEDGSVQLADHYQQNTPIGDGPVLLPDNH YLSTQSVLSKDPNEKRDHMVLLEFVTAAGITHGMDELYK
p15GFP	MGASKGERLFTGVVPILVELDGDVNGHKFSVRGEGEGDATRGKLTLKFIC TTGKLPVPWPTLVTTLTYGVQCFSRYPKHMKRHDFFKSAMPEGYVQERTI SFKKDGTYKTRAEVKFEGRTLVNRIELKGRDFKEKGNILGHKLEYNFNSH NVYITADKRKNGIKANFKIRHNVKDGSVQLADHYQQNTPIGRGPVLLPRN HYLSTRSALSKDPKEKRDHMVLLEFVTAAGITHGMDELYK
p15GFP Kv	MGASKGEKLFTGVVPILVELDGDVNGHKFSVRGEGEGDATKGKLTLKFIC TTGKLPVPWPTLVTTLTYGVQCFSRYPKHMKRHDFFKSAMPEGYVQERTI SFKKDGTYKTRAEVKFEGKTLVNRIELKGKDFKEKGNILGHKLEYNFNSH NVYITADKKKNGIKANFKIRHNVKDGSVQLADHYQQNTPIGKGPVLLPKN HYLSTKSALSKDPKEKRDHMVLLEFVTAAGITHGMDELYK
n20GFP	MGASKGEELFTGVVPILVELDGDVNGHKFSVRGEGEGDATNGKLTLKFIC TTGKLPVPWPTLVTTLTYGVQCFSRYPDHMDQHDFFKSAMPEGYVQERTI SFKDDGTYKTRAEVKFEGDTLVNRIELKGIDFKEDGNILGHKLEYNFNSHD VYITADKQENGIKAEFEIRHNVEDGSVQLADHYQQNTPIGDGPVLLPDDH YLSTESALSKDPNEDRDHMVLLEFVTAAGIDHGMDELYK

Table S3.
Sequence information for Sensor A and dTEVp-based client.

Construct	Sequence
Sensor A	MGASKGERLFTGVVPILVELDGDVNGHKFSVRGEGEGDATRGKLTLKFIC<br>TTGKLPVPWPTLVTTLTYGVQCFSRYPKHMKRHDFFKSAMPEGYVQERTI<br>SFKKDGTYKTRAEVKFEGRTLVNRIELKGRDFKEKGNILGHKLEYNFNSH<br>NVYITADKRKNGIKANFKIRHNVKDGSVQLADHYQQNTPIGRGPVLLPRN<br>HYLSTRSALSKDPKEKRDHMVLLEFVTAAGITHGMDELYKSGLELLEDLT<br>LGSPGYLFSGSHGSTEGSRGRASEQYSQQHSGAQGHASVSQTDSDESHGE<br>NDSAQSGSYGRHGSDRVASSAQGHGIGSHRSSDASQQYHSGYREGASSRA<br>QEHSSAAQGGSSTQTYRTGSQRSSDASYGHGARDEQHSRPYRSGDRLQEQ<br>ASGHHNRSASGSWRESDESHGESDSDQSVSSGQRRSRRSGESQGQVAQSY<br>RSIDAHTSAEYHRSGSYRSGDGSQEENRTTRSASRSGSQGRSADSRGQSTT<br>HSHYSGSYGSSDVSHEYYYGQRGGRSSGPYGRITDQGDQSASYRSSDQAQ<br>EYRSGQRGRTSPGTWGYSSESQEYTSVQGS
sfGFP-dTEVp	MGSKGEELFTGVVPILVELDGDVNGHKFSVRGEGEGDATNGKLTLKFICT<br>TGKLPVPWPTLVTTLGYGVQCFSRYPDHMKRHDFFKSAMPEGYVQERTIS<br>FKDDGTYKTRAEVKFEGDTLVNRIELKGIDFKEDGNILGHKLEYNFNSHN<br>VYITADKQKNGIKANFKIRHNVEDGSVQLADHYQQNTPIGDGPVLLPDNH<br>YLSTQSVLSKDPNEKRDHMVLLEFVTAAGITHGMDELYKSPGSPGSGESLF<br>KGPRDYNPISSTICHLTNESDGHTTSLYGIGFGPFIITNKHLFRRNNGTLLVQ<br>SLHGVFKVKNTTTLQQHLIDGRDMIIIRMPKDFPPFPQKLKFREPQREERIC<br>LVTTNFQTKSMSSMVSDTSCTFPSSDGIFWKHWIQTKDGQAGSPLVSTRD<br>GFIVGIHSASNFTNTNNYFTSVPKNFMELLTNQEAQQWVSGWRLNADSVL<br>WGGHKVFMVKPEEPFQPVKEATQLMNELVYSQ

Table S4.
Sequence information for IDP-scaffolds (+Pro from P2A cleavage) in Fig. 6.

Construct	Sequence
**S1**	PASSEDVIKEFMRFKVRMEGSVNGHEFEIEGEGEGRPYEGTQTAKLKVTK<br>GGPLPFAWDILSPQFQYGSKAYVKHPADIPDYLKLSFPEGFKWERVMNFE<br>DGGVVTVTQDSSLQDGEFIYKVKLRGTNFPSDGPVMQKKTMGWEASTER<br>MYPEDGALKGEIKMRLKLKDGGHYDAEVKTTYMAKKPVQLPGAYKTDI<br>KLDITSHNEDYTIVEQYERAEGRHSTGASGSENLYFQSGPGGQVSTHEQSE<br>SSHGWTGPSTRGRQGSRHEQAQDSSRHSASQDGQDTIRGHPGSSRGGRQG<br>YHHEHSVDSSGHSGSHHSHTTSQGRSDASRGQSGSRSASRTTRNEEQSGD<br>GSRHSGSRHHEASTHADISRHSQAVQGQSEGSRRSRRQGSSVSQDSDSEG<br>HSEDSERWSGSASRNHHGSAQEQLRDGSRHPRSHQEDRAGHGHSADSSR<br>QSGTRHTQTSSGGQAASSHEQARSSAGERHGSHHQQSADSSRHSGIGHGQ<br>ASSAVRDSGHRGYSGSQASDNEGHSEDSDTQSVSAHGQAGSHQQSHQES<br>ARGRSGETSGHSGSFLYGQVSTHEQSESSHGWTGPSTRGRQGSRHEQAQD<br>SSRHSASQDGQDTIRGHPGSSRGGRQGYHHEHSVDSSGHSGSHHSHTTSQ<br>GRSDASRGQSGSRSASRTTRNEEQSGDGSRHSGSRHHEASTHADISRHSQA<br>VQGQSEGSRRSRRQGSSVSQDSDSEGHSEDSERWSGSASRNHHGSAQEQL<br>RDGSRHPRSHQEDRAGHGHSADSSRQSGTRHTQTSSGGQAASSHEQARSS<br>AGERHGSHHQQSADSSRHSGIGHGQASSAVRDSGHRGYSGSQASDNEGH<br>SEDSDTQSVSAHGQAGSHQQSHQESARGRSGETSGHSGSFLYGENLYFQS<br>VPGSGQVSTHEQSESSHGWTGPSTRGRQGSRHEQAQDSSRHSASQDGQDT<br>IRGHPGSSRGGRQGYHHEHSVDSSGHSGSHHSHTTSQGRSDASRGQSGSR<br>SASRTTRNEEQSGDGSRHSGSRHHEASTHADISRHSQAVQGQSEGSRRSRR<br>QGSSVSQDSDSEGHSEDSERWSGSASRNHHGSAQEQLRDGSRHPRSHQED<br>RAGHGHSADSSRQSGTRHTQTSSGGQAASSHEQARSSAGERHGSHHQQS<br>ADSSRHSGIGHGQASSAVRDSGHRGYSGSQASDNEGHSEDSDTQSVSAHG<br>QAGSHQQSHQESARGRSGETSGHSGSFLYGQVSTHEQSESSHGWTGPSTR<br>GRQGSRHEQAQDSSRHSASQDGQDTIRGHPGSSRGGRQGYHHEHSVDSS<br>GHSGSHHSHTTSQGRSDASRGQSGSRSASRTTRNEEQSGDGSRHSGSRHH<br>EASTHADISRHSQAVQGQSEGSRRSRRQGSSVSQDSDSEGHSEDSERWSGS<br>ASRNHHGSAQEQLRDGSRHPRSHQEDRAGHGHSADSSRQSGTRHTQTSS<br>GGQAASSHEQARSSAGERHGSHHQQSADSSRHSGIGHGQASSAVRDSGH<br>RGYSGSQASDNEGHSEDSDTQSVSAHGQAGSHQQSHQESARGRSGETSG<br>HSGSFLYGENLYFQSVPGSGPGLCGHSSDISKQLGFSQSQRYYYYEG
**S2**	PASSEDVIKEFMRFKVRMEGSVNGHEFEIEGEGEGRPYEGTQTAKLKVTK<br>GGPLPFAWDILSPQFQYGSKAYVKHPADIPDYLKLSFPEGFKWERVMNFE<br>DGGVVTVTQDSSLQDGEFIYKVKLRGTNFPSDGPVMQKKTMGWEASTER<br>MYPEDGALKGEIKMRLKLKDGGHYDAEVKTTYMAKKPVQLPGAYKTDI<br>KLDITSHNEDYTIVEQYERAEGRHSTGASGSENLYFQSGPGGQVSTHEQSE<br>SSHGWTGPSTRGRQGSRHEQAQDSSRHSASQDGQDTIRGHPGSSRGGRQG<br>YHHEHSVDSSGHSGSHHSHTTSQGRSDASRGQSGSRSASRTTRNEEQSGD

	GSRHSGSRHHEASTHADISRHSQAVQGQSEGSRRSRRQGSSVSQDSDSEG<br>HSEDSERWSGSASRNHHGSAQEQLRDGSRHPRSHQEDRAGHGHSADSSR<br>QSGTRHTQTSSGGQAASSHEQARSSAGERHGSHHQQSADSSRHSGIGHGQ<br>ASSAVRDSGHRGYSGSQASDNEGHSEDSDTQSVSAHGQAGSHQQSHQES<br>ARGRSGETSGHSGSFLYGQVSTHEQSESSHGWTGPSTRGRQGSRHEQAQD<br>SSRHSASQDGQDTIRGHPGSSRGGRQGYHHEHSVDSSGHSGSHHSHTTSQ<br>GRSDASRGQSGSRSASRTTRNEEQSGDGSRHSGSRHHEASTHADISRHSQA<br>VQGQSEGSRRSRRQGSSVSQDSDSEGHSEDSERWSGSASRNHHGSAQEQL<br>RDGSRHPRSHQEDRAGHGHSADSSRQSGTRHTQTSSGGQAASSHEQARSS<br>AGERHGSHHQQSADSSRHSGIGHGQASSAVRDSGHRGYSGSQASDNEGH<br>SEDSDTQSVSAHGQAGSHQQSHQESARGRSGETSGHSGSFLYGQVSTHEQ<br>SESSHGWTGPSTRGRQGSRHEQAQDSSRHSASQDGQDTIRGHPGSSRGGR<br>QGYHHEHSVDSSGHSGSHHSHTTSQGRSDASRGQSGSRSASRTTRNEEQS<br>GDGSRHSGSRHHEASTHADISRHSQAVQGQSEGSRRSRRQGSSVSQDSDS<br>EGHSEDSERWSGSASRNHHGSAQEQLRDGSRHPRSHQEDRAGHGHSADS<br>SRQSGTRHTQTSSGGQAASSHEQARSSAGERHGSHHQQSADSSRHSGIGH<br>GQASSAVRDSGHRGYSGSQASDNEGHSEDSDTQSVSAHGQAGSHQQSHQ<br>ESARGRSGETSGHSGSFLYGQVSTHEQSESSHGWTGPSTRGRQGSRHEQA<br>QDSSRHSASQDGQDTIRGHPGSSRGGRQGYHHEHSVDSSGHSGSHHSHTT<br>SQGRSDASRGQSGSRSASRTTRNEEQSGDGSRHSGSRHHEASTHADISRHS<br>QAVQGQSEGSRRSRRQGSSVSQDSDSEGHSEDSERWSGSASRNHHGSAQE<br>QLRDGSRHPRSHQEDRAGHGHSADSSRQSGTRHTQTSSGGQAASSHEQA<br>RSSAGERHGSHHQQSADSSRHSGIGHGQASSAVRDSGHRGYSGSQASDNE<br>GHSEDSDTQSVSAHGQAGSHQQSHQESARGRSGETSGHSGSFLYGPGLCG<br>HSSDISKQLGFSQSQRYYYYEG
**S3**	PASSEDVIKEFMRFKVRMEGSVNGHEFEIEGEGEGRPYEGTQTAKLKVTK<br>GGPLPFAWDILSPQFQYGSKAYVKHPADIPDYLKLSFPEGFKWERVMNFE<br>DGGVVTVTQDSSLQDGEFIYKVKLRGTNFPSDGPVMQKKTMGWEASTER<br>MYPEDGALKGEIKMRLKLKDGGHYDAEVKTTYMAKKPVQLPGAYKTDI<br>KLDITSHNEDYTIVEQYERAEGRHSTGASGSENLYFQSGPGGQVSTHEQSE<br>SSHGWTGPSTRGRQGSRHEQAQDSSRHSASQDGQDTIRGHPGSSRGGRQG<br>YHHEHSVDSSGHSGSHHSHTTSQGRSDASRGQSGSRSASRTTRNEEQSGD<br>GSRHSGSRHHEASTHADISRHSQAVQGQSEGSRRSRRQGSSVSQDSDSEG<br>HSEDSERWSGSASRNHHGSAQEQLRDGSRHPRSHQEDRAGHGHSADSSR<br>QSGTRHTQTSSGGQAASSHEQARSSAGERHGSHHQQSADSSRHSGIGHGQ<br>ASSAVRDSGHRGYSGSQASDNEGHSEDSDTQSVSAHGQAGSHQQSHQES<br>ARGRSGETSGHSGSFLYGQVSTHEQSESSHGWTGPSTRGRQGSRHEQAQD<br>SSRHSASQDGQDTIRGHPGSSRGGRQGYHHEHSVDSSGHSGSHHSHTTSQ<br>GRSDASRGQSGSRSASRTTRNEEQSGDGSRHSGSRHHEASTHADISRHSQA<br>VQGQSEGSRRSRRQGSSVSQDSDSEGHSEDSERWSGSASRNHHGSAQEQL<br>RDGSRHPRSHQEDRAGHGHSADSSRQSGTRHTQTSSGGQAASSHEQARSS<br>AGERHGSHHQQSADSSRHSGIGHGQASSAVRDSGHRGYSGSQASDNEGH<br>SEDSDTQSVSAHGQAGSHQQSHQESARGRSGETSGHSGSFLYGQVSTHEQ<br>SESSHGWTGPSTRGRQGSRHEQAQDSSRHSASQDGQDTIRGHPGSSRGGR<br>QGYHHEHSVDSSGHSGSHHSHTTSQGRSDASRGQSGSRSASRTTRNEEQS<br>GDGSRHSGSRHHEASTHADISRHSQAVQGQSEGSRRSRRQGSSVSQDSDS

	EGHSEDSERWSGSASRNHHGSAQEQLRDGSRHPRSHQEDRAGHGHSADS<br>SRQSGTRHTQTSSGGQAASSHEQARSSAGERHGSHHQQSADSSRHSGIGH<br>GQASSAVRDSGHRGYSGSQASDNEGHSEDSDTQSVSAHGQAGSHQQSHQ<br>ESARGRSGETSGHSGSFLYGQVSTHEQSESSHGWTGPSTRGRQGSRHEQA<br>QDSSRHSASQDGQDTIRGHPGSSRGGRQGYHHEHSVDSSGHSGSHHSHTT<br>SQGRSDASRGQSGSRSASRTTRNEEQSGDGSRHSGSRHHEASTHADISRHS<br>QAVQGQSEGSRRSRRQGSSVSQDSDSEGHSEDSERWSGSASRNHHGSAQE<br>QLRDGSRHPRSHQEDRAGHGHSADSSRQSGTRHTQTSSGGQAASSHEQA<br>RSSAGERHGSHHQQSADSSRHSGIGHGQASSAVRDSGHRGYSGSQASDNE<br>GHSEDSDTQSVSAHGQAGSHQQSHQESARGRSGETSGHSGSFLYGQVSTH<br>EQSESSHGWTGPSTRGRQGSRHEQAQDSSRHSASQDGQDTIRGHPGSSRG<br>GRQGYHHEHSVDSSGHSGSHHSHTTSQGRSDASRGQSGSRSASRTTRNEE<br>QSGDGSRHSGSRHHEASTHADISRHSQAVQGQSEGSRRSRRQGSSVSQDS<br>DSEGHSEDSERWSGSASRNHHGSAQEQLRDGSRHPRSHQEDRAGHGHSA<br>DSSRQSGTRHTQTSSGGQAASSHEQARSSAGERHGSHHQQSADSSRHSGI<br>GHGQASSAVRDSGHRGYSGSQASDNEGHSEDSDTQSVSAHGQAGSHQQS<br>HQESARGRSGETSGHSGSFLYGQVSTHEQSESSHGWTGPSTRGRQGSRHE<br>QAQDSSRHSASQDGQDTIRGHPGSSRGGRQGYHHEHSVDSSGHSGSHHSH<br>TTSQGRSDASRGQSGSRSASRTTRNEEQSGDGSRHSGSRHHEASTHADISR<br>HSQAVQGQSEGSRRSRRQGSSVSQDSDSEGHSEDSERWSGSASRNHHGSA<br>QEQLRDGSRHPRSHQEDRAGHGHSADSSRQSGTRHTQTSSGGQAASSHEQ<br>ARSSAGERHGSHHQQSADSSRHSGIGHGQASSAVRDSGHRGYSGSQASDN<br>EGHSEDSDTQSVSAHGQAGSHQQSHQESARGRSGETSGHSGSFLYGQVST<br>HEQSESSHGWTGPSTRGRQGSRHEQAQDSSRHSASQDGQDTIRGHPGSSR<br>GGRQGYHHEHSVDSSGHSGSHHSHTTSQGRSDASRGQSGSRSASRTTRNE<br>EQSGDGSRHSGSRHHEASTHADISRHSQAVQGQSEGSRRSRRQGSSVSQD<br>SDSEGHSEDSERWSGSASRNHHGSAQEQLRDGSRHPRSHQEDRAGHGHS<br>ADSSRQSGTRHTQTSSGGQAASSHEQARSSAGERHGSHHQQSADSSRHSG<br>IGHGQASSAVRDSGHRGYSGSQASDNEGHSEDSDTQSVSAHGQAGSHQQ<br>SHQESARGRSGETSGHSGSFLYGQVSTHEQSESSHGWTGPSTRGRQGSRH<br>EQAQDSSRHSASQDGQDTIRGHPGSSRGGRQGYHHEHSVDSSGHSGSHHS<br>HTTSQGRSDASRGQSGSRSASRTTRNEEQSGDGSRHSGSRHHEASTHADIS<br>RHSQAVQGQSEGSRRSRRQGSSVSQDSDSEGHSEDSERWSGSASRNHHGS<br>AQEQLRDGSRHPRSHQEDRAGHGHSADSSRQSGTRHTQTSSGGQAASSHE<br>QARSSAGERHGSHHQQSADSSRHSGIGHGQASSAVRDSGHRGYSGSQASD<br>NEGHSEDSDTQSVSAHGQAGSHQQSHQESARGRSGETSGHSGSFLYGPGL<br>CGHSSDISKQLGFSQSQRYYYYEG
